# The role of kaolin and kaolin/ZnO nanoadsorbents in adsorption studies for tannery wastewater treatment

**DOI:** 10.1038/s41598-020-69808-z

**Published:** 2020-08-03

**Authors:** S. Mustapha, J. O. Tijani, M. M. Ndamitso, S. A. Abdulkareem, D. T. Shuaib, A. K. Mohammed, A. Sumaila

**Affiliations:** 1 Department of Chemistry, Federal University of Technology, Bosso Campus, PMB 65, Minna, Nigeria; 2 Department of Chemical Engineering, Federal University of Technology, GidanKwano Campus, PMB 65, Minna, Niger State Nigeria; 3 Nanotechnology Research Group, Africa Center of Excellence for Mycotoxin and Food Safety, Federal University of Technology, PMB 65, Minna, Niger State Nigeria; 4 Department of Chemistry, Illinois Institute of Technology, 3101 S Dearborn Street, Chicago, IL 60616 USA; 5 Department of Chemistry and Biochemistry, North Carolina Central University, 1801 Fayetteville Street, Durham, NC 27707 USA; 6 Department of Pure and Industrial Chemistry, Anyigba, Kogi State Nigeria

**Keywords:** Natural hazards, Nanoscale materials

## Abstract

In the present study, comparative studies of kaolin and kaolin/ZnO nanocomposites for the adsorption of Cr(VI), Fe(III), COD, BOD, and chloride from tannery wastewater were investigated. ZnO nanoparticles and kaolin/ZnO nanocomposites were prepared by sol–gel followed by wet-impregnation methods. The prepared adsorbents were characterized using different analytical tools such as X-ray diffraction, Fourier transforms infrared, high-resolution transmission electron microscopy, energy dispersive spectroscopy, selective area electron diffraction and Brunauer Emmett–Teller (BET) and X-ray Photoelectron Spectroscopy (XPS). The HRSEM/EDS/XPS analysis confirmed successful immobilization of clay structural network on the lattice layers of zincite hexagonal structure of ZnO nanoparticles. BET measurement showed an increase in the surface area of kaolin/ZnO nanocomposites (31.8 m^2^/g) when compared to kaolin (17 m^2^/g). Batch adsorption studies were carried out by varying the parameters such as contact time, adsorbent dosage and temperature. The maximum removal of Cr(VI) (100%), Fe(III) (98%), COD (95%), BOD (94%) and Chloride (78%) was obtained at 15 min by kaolin/ZnO composites. While 78% Cr(VI), 91% Fe(III), 91% COD, 89% BOD and 73% Chloride were removed by kaolin under the same conditions. The kaolin/ZnO nanocomposites exhibited better adsorption performance than kaolin due to higher surface area of the former than the latter. It was found that the Jovanovic isotherm model fitted the adsorption experimental data most with the highest correlation (R^2^ > 0.99) for both nanoadsorbents and indicate the occurrence of adsorption on monolayer and heterogeneous surfaces. The mechanism for the adsorption of metal ions in tannery wastewater onto the nano-adsorbents was examined using Weber Morris intra-particle diffusion model and Boyd plot which showed that the adsorption process was both intra-particle and film diffusion controlled. The thermodynamic parameters such as enthalpy change showed that that adsorption of metal ions and other parameters was feasible, spontaneous and endothermic. The ZnO/clay nanocomposites exhibited excellent recyclable and re-useable properties even after six repeated applications and can, therefore, be applied in wastewater treatment for removal of heavy metals and other physicochemical parameters.

## Introduction

Environmental contamination via natural and anthropogenic activities have been recognized as one of the major global problems confronting the human race and aquatic species. Industrial manufacturing processes utilize a high amount of clean water during operations and at the same time generate and discharge a large volume of untreated wastewater into water bodies. Specifically, industrial tanning process which transforms animal hides and skins into leather generate highly turbid, coloured and foul-smelling wastewater containing different organic and inorganic pollutants especially chromium complex collagens, sulphides, chlorides, hydrogen sulphide, which have become threats to human and need to be addressed. Most of the chemicals used during pre-tanning and tanning operations include lime, ammonium sulphate, sodium chloride, chromium sulphate, sulphuric acid and sodium bicarbonate. One of the major heavy metals present in tannery wastewater is chromium and exists in two different forms trivalent (Cr^3+^ or Cr(III)), and hexavalent, (Cr^6+^ or Cr(VI)). Cr (III) is regarded as an essential trace element while Cr^6+^ has been reported to be more toxic to animals and non-essential than Cr^3+^ due to its high solubility in water and soil^1^. Exposure to wastewater containing Cr(VI) can lead to dermatitis, occupational asthma, eye irritation, kidney and liver damage, lung cancer, respiratory irritation pulmonary congestion, oedema, skin irritation amongst others. The Cr(VI) can easily bioaccumulate along food chains and eventually result in biomagnifications and therefore become a major threat to human and aquatic species^2^. Also, Fe exists in several oxidation states such as + 2, + 3, + 4 and + 6 and exposure to wastewater containing a high concentration of Fe can result in chronic fatigue, joint pain, diabetics mellitus, heart attack, and liver disease to mention but a few^3^. It is obvious that tannery wastewater represents an important source of environmental contamination and delay in treatment can lead to different health challenges since it is toxic and contain several non-biodegradable constituents.

In recent years, environmental pollution control mechanism as related to wastewater has become a topical issue among environmentalist owing to its negative influence the pollutants on human^4^. Thus, water pollution is a menace that causes environmental degradation, reducing the water quality and therefore becomes imperative to treat tannery wastewater before releasing into the environment to alleviate the adverse effects of toxic pollutants on human and aquatic life.

Different conventional techniques for the removal of pollutants from wastewater include solvent extraction, ion exchange, chemical reduction, precipitation, electrolysis, electro-dialysis, micro and ultra-filtration, reverse osmosis, advanced oxidation process and membrane filtration^5,6^. However, these conventional methods have some disadvantages such as lower efficiency and high costs which discourage many industries from adopting any treatment methods. Studies on the treatment of wastewater have revealed adsorption platform with nanotechnology to be one of the most effective techniques for the removal of pollutants from wastewater^7,8^. This method is based on the imbalance of attractive forces of atoms on the surface of adsorbents. Adsorption is found to be a more efficient, eco-friendly, and cost-effective method for the removal of pollutants from wastewater due to its flexibility in design and ease of operation^2^. In recent years, different adsorbents such as zeolites, goethite, graphene oxide, activated carbon, agricultural wastes/byproducts, industrial wastes, metals/metal oxides nanomaterials and many more were successfully used to remove the pollutants from wastewater. Among these adsorbents is zinc oxide nanoparticles (ZnONPs), an n-type semiconductor metal oxide which exhibits excellent adsorption and photocatalytic activity in the absence or presence of sunlight^9^. It is considered non-toxic, has a high resistance to chemical and optical corrosion, high catalytic activity and stable chemical properties^10,11,12^. ZnONPs prepared from precursors such as zinc acetate dihydrate, zinc nitrate hexahydrate, zinc chloride, zinc sulphate and zinc acetylacetonate using physical or chemical methods are either in pure form or anchored on a suitable matrix. The drawbacks of ZnONPs include low surface area, rapid agglomeration and small particle size, which makes recovery from the aqueous phase very difficult. Additionally, the discharge of ZnONPs into the environment could pose potential health and environmental risks. Therefore, the immobilization of ZnONPs on a suitable matrix could help to tackle these problems^13^. The supported nanoparticles usually possess excellent adsorption capacity, mechanical properties, thermal stability and high specific surface area compared to non-supported metal oxide nanoparticles. Clay is very suitable for this purpose due to its low cost, and wide availability. Additionally, their structures determine useful physical and chemical properties such as specific surface area, water hold capacity, ion exchange capacity and reactivity^14,15,16^. The exchangeable ions help to scavenge natural pollutants in wastewater via adsorption and ion exchange. However, cost should be an important parameter to be considered for their application. Thus, the need to know the performance of the adsorption process for pollutants removal in wastewater under various experimental condition is very important. Therefore, there is an urgent need to thoroughly investigate the feasibility of using kaolin and hybrid clay based on kaolinite clay and ZnO-NPs as for the adsorption of pollutants from wastewater.

Until now, a very limited effort has been made to introduce ZnO-NPs on the surface of clay minerals to prove their potential usefulness as nanoadsorbents for the removal of emerging contaminants from industrial wastewater. For instance, Azizi et al.^17^ employed green synthesized ZnO nanoparticles to remove Pb(II) ion from aqueous solution and achieved maximum adsorption capacity and removal efficiency of 19.65 mg/g and 93% respectively under the following conditions: pH (5), initial concentration of Pb(II) (10 mg/L) temperature (70 °C), dosage (0.1 g), contact time (1 h), solution volume (25 mL). Zolfaghari et al.^18^ have found that that higher adsorption capacity of 2.52 mmol/g for ZnO/coated nanoporous carbon was responsible for 90% Pb(II) removal from the aqueous solution compared to ZnO alone with 2.38 mmol/g and 78% removal efficiency under the applied conditions. Salehi-Babarsad et al.^19^ employed have applied ZnO/polyaniline and ZnO/graphene oxide composites for the removal of heavy metal ions, such as Cr^6+^, Cr^3+^, Cu^2+^ and Fe^3+^ in aqueous solution. The authors established that support materials (polyaniline and graphene oxide) contributed to the high adsorptive capacity of ZnO nanoparticles. In the same vein, Sonawane et al.^20^ reported that ZnO/bentonite was efficient for the photocatalytic degradation of safranine in aqueous media however failed to investigate the adsorption potentials of the material. The removal of lead and copper ions from simulated wastewater using ZnO/montmorillonite nanocomposites was investigated by Sani et al.^4^. It was found that the adsorption capacity and surface areas of ZnO-NPs was enhanced by the addition of clay. Also, ZnO/clay nanocomposites prepared via impregnation method over alginate beads were used for the adsorption of congo red dye from simulated effluents^21^. The authors found the synthesized ZnO/clay material had high adsorption capacity and was attributed to the presence of ZnO-NPs in the intercalated nanocomposites. Thus, the removal of pollutants in simulated wastewater by nanocomposites was found to be excellent but there is a need for further clarification on the adsorption mechanism of the nanoadsorbents for pollutants present in the complex industrial wastewater. Moreso, Yu et al.^22^ found that biochar loaded with 30% ZnO nanoparticles removed 95% Cr(VI) from aqueous solution compared to biochar impregnated with 10% ZnO composites with 85% Cr(VI) removal efficiency after 14 h. Kamaraj et al.^9^ utilized ZnO and ZnO/activated carbon composites as nano-adsorbent and photocatalyst to degrade and remove methylene blue and Cr(VI) in aqueous solution and real industrial tannery wastewater. The authors found that ZnO/activated carbon composites exhibited improved and better removal efficiency of Cr(VI) and methylene blue than ZnO nanoparticles alone due to combined mechanism, high surface area, and surface functionality.

To date the scientific literature on the use of ZnO/kaolin nanocomposites for the treatment of pollutants in the tannery wastewater are very scarce, hence, this study focused on the removal of selected heavy metals (Cr and Fe) and other water quality indicator parameters (COD, BOD, and chloride) onto kaolin and kaolin/ZnO nanocomposites. The ZnO/kaolin nanocomposites were prepared by combination of sol–gel and wet-impregnation methods and subsequently characterized by different analytical tools. The batch adsorption behaviour of the materials via variation of different operating parameters such as contact time, temperature and adsorbent dosage were investigated. The adsorption rate isotherms, kinetics and thermodynamics for these aforementioned pollutants onto kaolin and hybrid clay were also investigated, and the stability and recyclability potentials of the hybrid materials were also studied.

## Materials and methods

### Materials

The raw kaolin used in this study was obtained from a deposit in Gbako Local Government Area in Niger State, Nigeria located at longitude and latitude at 9°24′00″N 6°02′00″E, respectively. Zinc acetate dihydrate [Zn(C_2_H_3_O_2_)_2_·2H_2_O], sodium hydroxide [NaOH (≥ 97%)], ethanol [C_2_H_5_OH (≥ 97%)] and hydrochloric acid [HCl (37%)] were purchased from Sigma Aldrich and used without further purification.

### Sampling and characterization of tannery wastewater

The tannery wastewater sample was collected in a virgin 2000 cm^3^ polyethene bottle from a Majematannery Industry, located at Manuri Road, Tudun Wada Area, Sokoto State, Nigeria and immediately transported to the laboratory prior for analysis. The physicochemical parameters of the wastewater samples such as pH, colour, biochemical oxygen demand (BOD), chemical oxygen demand (COD), chloride, sulphate, nitrate, turbidity, electrical conductivity, total dissolved solids (TSS) and total suspended solids (TSS) were determined according to procedures described by the American Public Health Association (APHA)^23^. Heavy metals such as iron (Fe) and chromium (Cr) were measured by Atomic Absorption Spectroscopy (AAS) (Perkin Elmer 200 Atomic Absorption Spectrophotometer).

### Treatment of raw kaolin

The kaolin sample was suspended in a 1,000 cm^3^ beaker filled with deionized water and stirred for 30 min for particle size screening. Afterwards, the suspension was allowed to settle for 24 h. Supernatant from the beaker was collected by dispersion, decantation and filtration techniques. The filtrate was oven-dried at 100 °C for 24 h until there was complete dryness. Further treatment was done on the kaolin as described by Mustapha et al.^24^. The dried sample was ground and then sieved prior to further analysis.

### Synthesis of ZnO nanoparticles

The zinc oxide nanoparticles were synthesized using the sol–gel method. 50 cm^3^ of 0.5 M Zn(C_2_H_3_O_2_)_2_·2H_2_O solution was measured into a 250 cm^3^ beaker and about 100.0 cm^3^ of de-ionized water was added. The solution was stirred using a magnetic stirrer at 150 rpm for 30 min. A solution of 0.5 M NaOH was added drop-wise to the Zn(C_2_H_3_O_2_)_2_·2H_2_O solution to obtain the desired pH. The mixture was stirred for 5 min and the precipitates obtained were filtered using a Whatmann No. 42 filter paper. The precipitates were washed with deionized water and ethanol to eliminate traces of the unreacted precursors. The final product was oven-dried at 105 °C for 24 h and finally calcined in the furnace at a temperature of 450 °C for 3 h to obtain ZnO nanoparticles.

### Synthesis of kaolin supported ZnO nanoparticles

**Figure 1:**
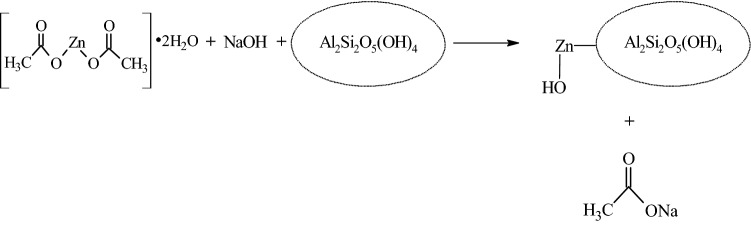
A schematic representation for preparation of kaolin/ZnO nanocomposites.

Combination of sol–gel and wet impregnation method was employed for the preparation of kaolin/ZnO as shown in Fig. 1. Zn(C_2_H_3_O_2_)_2_·2H_2_O (5.0 g) was first dissolved in de-ionized water (50.0 cm^3^) and stirred for 30 min, then sodium hydroxide solution (0.5 M) was added in drops into the precursor solution to adjust the pH to 6, 8, 10 and 12. To the white suspension formed, 0.5 g of the beneficiated clay was dispersed under vigorous stirring at 150 rpm for 1 h. A kaolin/ZnO homogeneous gel was formed and allowed to age overnight. The product was washed with de-ionized water and oven-dried at 80 °C overnight and finally calcined in the furnace at 450 °C for 3 h.

### Characterization of kaolin, ZnO nanoparticles and kaolin/ZnO nanocomposites

The identification of phases and the crystallite sizes of the synthesized nanoparticles and nanocomposites were determined by a Bruker AXS D8 Advance X-ray diffractometer with Cu Kα radiation. The powder samples were sprinkled on a de-greased glass slide and their diffraction pattern were recorded between diffraction angles of 20°–90°. The phase identifications were done by comparison with available d-spacing information and peaks from the Joint Committee on Powder Diffraction Standard (JCPDS). The morphologies of the synthesized samples were examined using a Zeiss Auriga HRSEM. About 0.05 mg of each sample was sprinkled onto carbon adhesives tape and sputter-coated with Au–Pd using a Quorum T15OT for 5 min. The microscope was operated with electron high tension at 5 kV for imaging. High resolution scanning electron microscopy (HRSEM) equipped with energy dispersive spectroscopy (EDS) was further used to determine the elemental composition of the synthesized nanoparticles and nanocomposites. The particle size and distribution pattern were analyzed by Zeiss Auriga High-Resolution Transmission Electron Microscopy (HRTEM) coupled with energy dispersive spectroscopy (EDS). The BET surface area and average pore volume distributions were obtained from the plot of the volume adsorbed (cm^3^/g STP) against relative pressure. The Brunauer–Emmett–Teller (BET) surface area, total pore volume and pore size were determined using a NovawinQuantachrome instrument. Fourier transform infrared spectra (FTIR) of the synthesized samples were recorded using Thermo Scientific Nicolet iS5 instrument with iD5 ATR spectrometer at a wavenumber range of 4,000–500 cm^−1^. X-ray photoelectron spectroscopy (XPS) model with monochromated Mg Kα (1,253.6 eV) operated at a power of 300 W and 15 eV were used to examine the surface elemental oxidation states of ZnO nanoparticles and kaolin/ZnO nanocomposites.

### Batch adsorption experiment

The adsorption experiments were performed by shaking a known weight of kaolin and kaolin/ZnO nanocomposites with 40 cm^3^ tannery wastewater solution in a 250 cm^3^ Erlenmeyer flask. Contact times ranging from 0 to 30 min were used to study the effect of time on adsorption of the pollutants in the solution and to attain the equilibrium time at 150 rpm. Furthermore, the effects of dosage (between 0.4 and 1.4 g) and temperature (30 °C and 80 °C) on the removal of pollutants from wastewater at their optimum time using speed rotation of 150 rpm were investigated. Supernatants were analyzed to determine the final concentration of some physicochemical parameters (COD, BOD and chloride) using APHA, manual method, 2005 and Atomic Absorption Spectrophotometer (AAS), (Perkin Elmer 200) respectively for Cr^6+^ and Fe^3+^. The various operating conditions of batch adsorption studies were carried out in duplicate. The percentage removal and adsorption capacity of the parameter by the adsorbents were calculated using Eqs. (1) and (2), respectively.

1 $$ \% {\text{ Removal}} = \frac{{{\text{C}}_{{\text{o}}} - {\text{C}}_{{\text{e}}} }}{{{\text{C}}_{{\text{o}}} }} \times 100 $$

2 $$ {\text{q}}_{{\text{e}}} = \frac{{\left( {{\text{C}}_{{\text{o}}} - {\text{C}}_{{\text{e}}} } \right)}}{{\text{M}}}{\text{V}} $$

where ${\text{C}}_{0}$ (mg/dm^3^) is the initial concentration of a pollutant in aqueous solution, ${\text{C}}_{{\text{e}}}$ (mg/dm^3^) is the concentration of pollutant aqueous solution at equilibrium, V (dm^3^) is the volume of the wastewater used in the experiment, and M (g) is the mass of the adsorbent.

## Results and discussion

### Characterization of tannery wastewater

The physical and chemical properties of the tannery wastewater were reported in our previous studies by Mustapha et al.^24^. The results showed that the indicator parameters in the wastewater exceeded the standard permissible limits established by the World Health Organization^25^ for industrial wastewater. This informed the decision to treat the tannery wastewater prior to discharge into the environment using kaolin and ZnO/kaolin nanocomposites as adsorbents.

### XRD analysis

**Figure 2:**
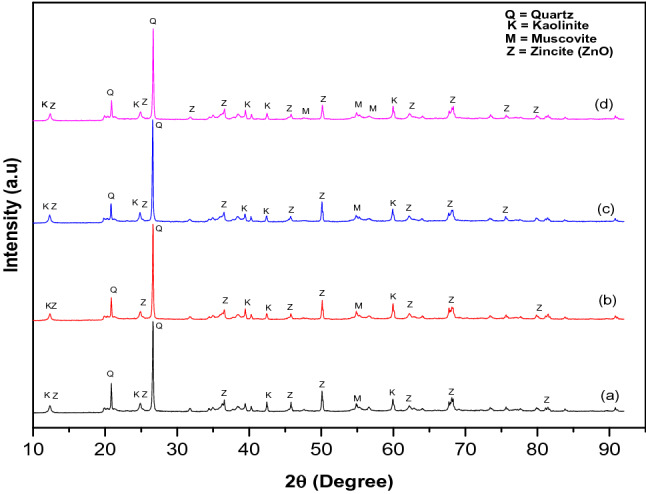
XRD pattern of kaolin/ZnO nanocomposites at pH (a) 6 (b) 8 (c) 10 and (d) 12 at calcination temperature of 450 °C for 3 h.

**Figure 3:**
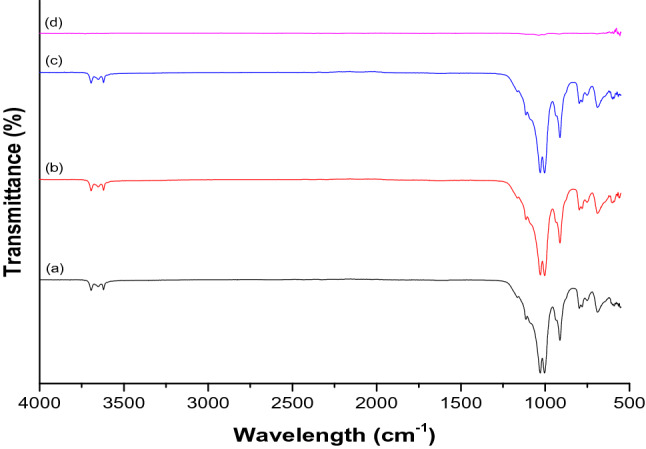
FTIR nanocomposites spectra of kaolin/ZnO at pH (a) 6 (b) 8 (c) 10 and (d) 12 calcined at 450 °C.

Figure 2 depicts the X-ray diffraction (XRD) pattern of the ZnO/kaolin nanocomposites prepared at pH (a) 6 (b) 8 (c) 10 and (d) 12. The ZnO nanoparticles loaded onto the matrix (kaolin) in the XRD pattern showed the presence of diffraction related to the zincite hexagonal structure irrespective of the pH. The low intensity of the diffraction peaks at 2θ values of 31.79°, 34.42°, 36.25° and 56.60° with corresponding Miller-Bravais indices (100), (002), (101), (102) and (110) crystal plane further confirmed the presence of ZnO nanoparticles in the kaolin nanocomposites samples. The diffraction peaks and corresponding crystal plans closely matched that the standard JCPDS file of zincite phase of ZnO nanoparticles (No. 36-1451). The phase of ZnO nanoparticles reported in this research is in good agreement with the previous studies^26,27^. The XRD pattern confirmed the effective formation of mixed-phase of kaolin/ZnO nanocomposites. Singh et al.^28^ also reported mixed phases of Fe_3_O_4_/ZnO composites prepared via co-precipitation method. Noticeably, there was no evidence of peaks broadening and shifting of diffraction peaks towards higher angles within the investigated pH range. This further showed that the prepared kaolin/ZnO composites were highly crystalline in nature. The low and high-intensity diffraction peaks at 12° and 26.8° indicated the presence of kaolinite in the various nanocomposite samples. Except for kaolin/ZnO nanocomposites prepared at pH 8 which showed the medium reflection of pure ZnO at 2 theta value of 25.1° and others revealed a mixture of zinc oxide and kaolinite. Therefore, the characteristic reflection planes of ZnO for kaolin/ZnO at pH 8 matches the HRTEM characterization shown in Fig. 3. The crystallite size of the ZnO of kaolin/ZnO nanocomposites prepared at different pH values calculated using Debye–Scherrer’s equation was 22.16 nm, 15.02 nm, 17.65 nm and 20.52 nm. The crystallize sizes reported in this study is greater than 10 nm obtained by Singh et al.^28^ prepared Fe_3_O_4_ doped ZnO nanoparticles using co-precipitation method. The differences in size may be ascribed to the method of synthesis.

### FTIR analysis

The FTIR spectra of kaolin/ZnO nanocomposites prepared at different pH values followed by calcination at 450 °C for 3 h are presented in Fig. 3. The spectra of the nanocomposites showed the characteristics absorption bands at around 3,626–3,696 cm^−1^ (OH vibration), 1,034 cm^−1^ (Si–O stretching vibration), 1,114, 797, 787, 757 and 607 cm^−1^ (Si–O), 914 cm^−1^ (Ti–O–Si), 775 and 522 cm^−1^ (Si–O–Al). The presence of Zn–O in the nanocomposites was confirmed by the absorption peak at 513 cm^−1^. It can be seen that the spectra of kaolin/ZnO nanocomposites prepared at pH 6, 8 and 10 did not differ in wavenumbers and have similar transparent bands values. This was attributed to the existence of strong interaction between kaolin and ZnO under the pH range studied. This further indicates the synergetic effect between the bonding characteristics of ZnO and kaolin samples. On the other hand, there were no visible absorption peaks in the spectrum of Kaolin/ZnO composites prepared at pH 12 shows weak absorption bands, which implied strong antagonistic effect between the two materials at pH 12 due to high concentration of hydroxyl ions. None formation of absorption bands at pH 12 may also be linked to strong electrostatic repulsion between Zn^2+^ and Si^4+^/Al^3+^ in the kaolin matrix. The peaks at 1,253 cm^−1^ are related to COO^−^ asymmetric and symmetric stretching while the peak at 944 cm^−1^ was assigned to Zn–O–H stretching. The presence of different absorption bands in the kaolin/ZnO nanocomposites prepared at pH 6, 8 and 12 will aid the binding of the adsorbate onto the adsorbents.

### HRTEM analysis

**Figure 4:**
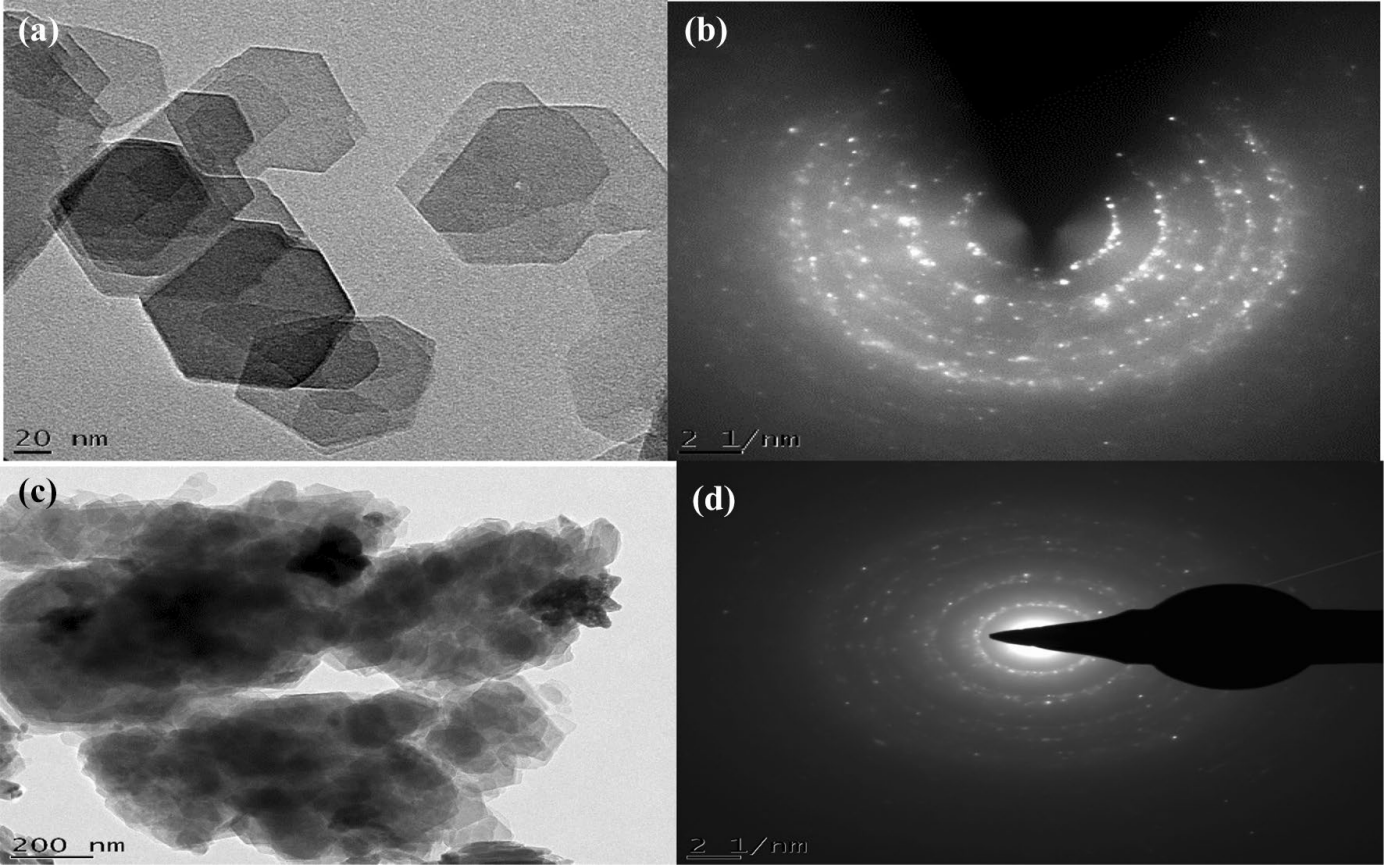
HRTEM and SAED analysis of beneficiated kaolin (**a**, **b**) and kaolin/ZnO (**c**, **d**) nanocomposites at pH of 8.

The HRTEM micrographs in Fig. 4a revealed the presence of less agglomerated but stacked hexagonal and plate-like shape of kaolin while Fig. 4b shows the presence of several diffractions and concentric ring pattern corresponding to each peak observed in the XRD pattern. shape ZnO nanoparticles homogeneously dispersed on the surface of the kaolin.

**Figure 5:**
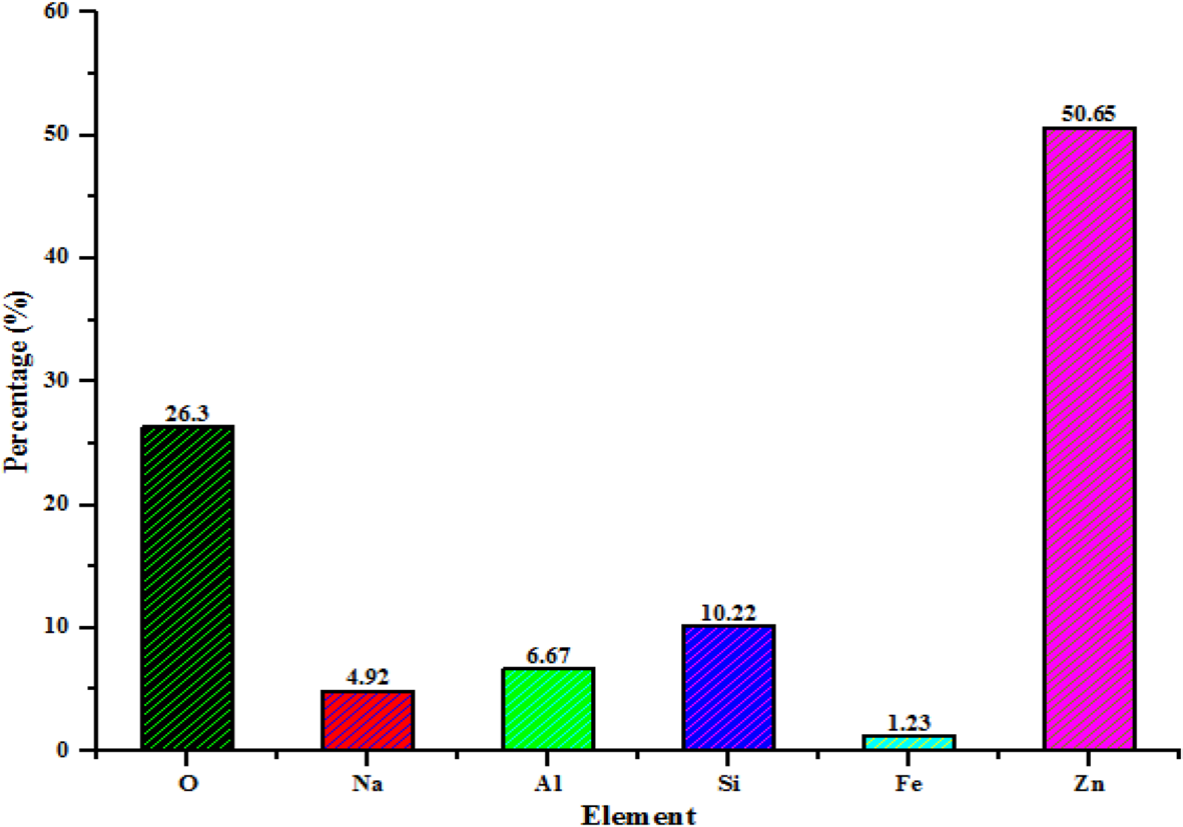
EDX analysis (atomic percentage) of kaolin/ZnO nanocomposites at a pH of 8.

HRTEM micrograph shown in Fig. 4c indicates successful coverage and deposition of spherical nanoparticles of ZnO on the silica-alumina interlayers spaces of kaolinites. The average size distribution of ZnO nanoparticles was within 21.34 nm. It was also noticed that the hexagonal and plate-like morphology of kaolin disappeared and transformed to spherical shapes in the composites. This shows that Zn with smaller ionic radius (139 pm) was responsible for the transformation of Si-Al dominant kaolinite network with an ionic radius of 210 pm and 184 pm respectively. Thus suggesting that the diffusion mechanism of Zn onto the kaolinite structures was responsible for the observed morphological changes. Figure 4c also indicates the presence of pores which aids the diffusion of metal ions and other pollutants during the adsorption process. The increase in porosity of kaolin/ZnO compared to kaolin alone as observed in HRTEM images may suggest higher removal efficiency of the pollutants by the former than the latter. The selective area electron diffraction (SAED) pattern of kaolin/ZnO nanocomposites (Fig. 4d) demonstrated the presence of a bright spot surrounded by sharp and intense concentric rings corresponding to the diffraction peaks noticed in the XRD pattern. Energy dispersive spectroscopy (EDX) analysis was carried out to confirm the presence and spatial distribution of Zn in the composites material. The EDX result shows the presence of Na, Al, O, Si, Fe and Zn elements (as seen in Fig. 5) in different amount. It can be seen that the dominant elements were Zn, O, Si and Al. Si and Al originated from kaolin while Zn and O were from the zinc salt precursors and other oxygen-containing species used for the synthesis. High percentage proportion of Zn^2+^ as shown in the EDX result implies evenly distribution of Zn in the composite matrix. While the EDX result suggests that other elements such as Si, Al were randomly impregnated into ZnO layers. A similar observation was reported by Singh et al.^28^ who embedded Fe_3_O_4_ onto lattice layers of ZnO nanoparticles. Thus, immobilization of ZnO nanoparticles on kaolin matrix prevents agglomeration of nanoparticles, leading to large and active surface sites of the nanocomposite for the adsorption process.

### BET analysis

**Figure 6:**
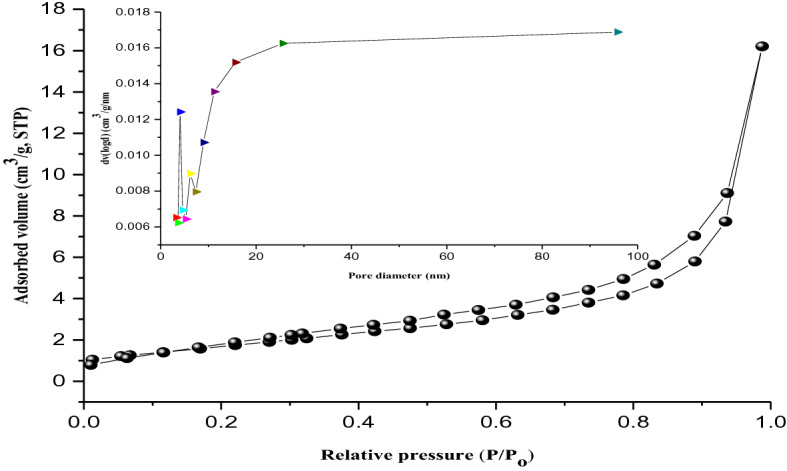
BET analysis for pore diameter (inlet) and surface area of kaolin.

**Figure 7:**
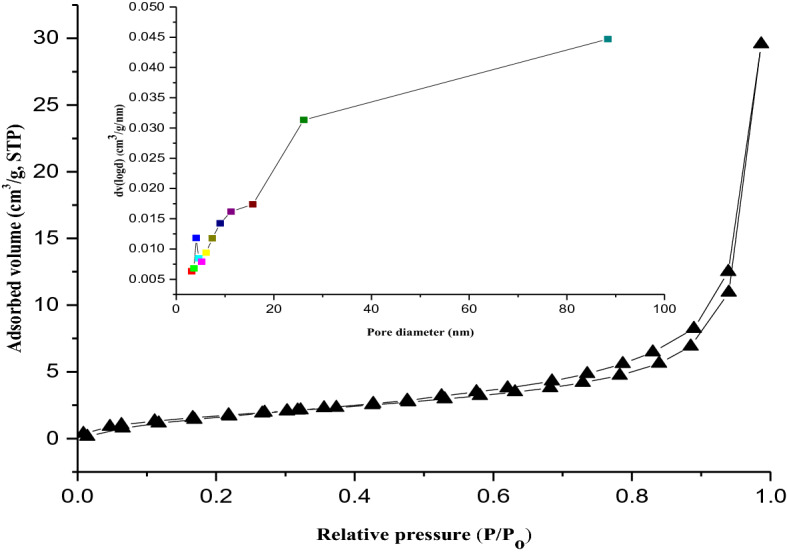
BET analysis for pore diameter (inlet) and surface area of kaolin/ZnO nanocomposites at a pH of 8.

The N_2_ adsorption–desorption isotherms of kaolin and kaolin/ZnO nanocomposites are shown in Figs. 6 and 7, respectively. The specific surface areas and pore diameter calculated by BET and Barret–Joyner Halenda methods for kaolin was 17 m^2^/g and 3.59 nm respectively. While that of kaolin/ZnO nanocomposites was 31.8 m^2^/g and 4.81 nm. It can be noticed that the surface area of kaolin/ZnO nanocomposites is much higher than that of kaolin alone, evidence of more binding sites in the former than the latter. One of the most important properties of adsorbent is surface area, the higher the surface area of kaolin/ZnO, the larger its adsorptive capacity. This suggests that the addition of porous and polar ZnO nanoparticles which further open the pores on kaolin was responsible for the increased surface area of the composites material. Also, Figs. 6 and 7, displayed a typical Type IV isotherm accompanied by Type H3 hysteresis loop according to IUPAC classification and suggest that the prepared adsorbents were predominantly mesoporous in nature^29^. However, the mesoporous dominant property was higher in kaolin than kaolin/ZnO nanocomposites due to wide pores opening in the former compared to the latter with closed pore opening till P/P_O_ of 0.7 (see Fig. 7). The differences in the extent of mesoporosity can be linked to the entrapment of ZnO nanoparticles by the kaolinite in kaolin/ZnO which block the pores compared to kaolin alone without ZnO nanoparticles. The prevalence of large mesopores in the two materials indicate that adsorption of N_2_ gas by these samples proceeds is multilayer in nature. It is also obvious that the capillary condensation of gases vis-a-viz the amount of adsorbed gases gradually increases with increasing relative pressure (P/P_o_) until approach unity. A closer look shows that the adsorption–desorption curve for both materials was stepwise and steeply slopy and more visible in kaolin than kaolin modified with ZnO composites. The pore size value strongly supports the finding that the kaolin/ZnO nanocomposites are mesoporous in nature. The pore volume vs pore diameter inserted in Fig. 7 revealed evenly distribution of ZnO nanoparticles onto the intercalated layers of kaolinite sample. The homogeneous distribution of ZnO nanoparticles may be linked to the slight decrease in the intensity of peaks observed in the range 5–100 nm for kaolin alone to 5–90 nm for kaolin/ZnO composites. The reduction in the pore size distribution of kaolin/ZnO composites compared to kaolin alone according to BJH curve may be due to entrapment of ZnO nanoparticles by kaolin and uncontrollable pore collapse of the kaolinite structure during calcination at 450 °C. It can also be observed the intensity of peak at 5 nm for kaolin in the BJH curve (Fig. 6) reduced after doping with ZnO nanoparticles (Fig. 7) is also an indication of more active sites in the latter than the former. Thus, modification of kaolin with ZnO nanoparticles causes a significant increase in the surface area compared to kaolin alone. A similar trend was reported by Singh et al.^28^.

### XPS analysis

**Figure 8:**
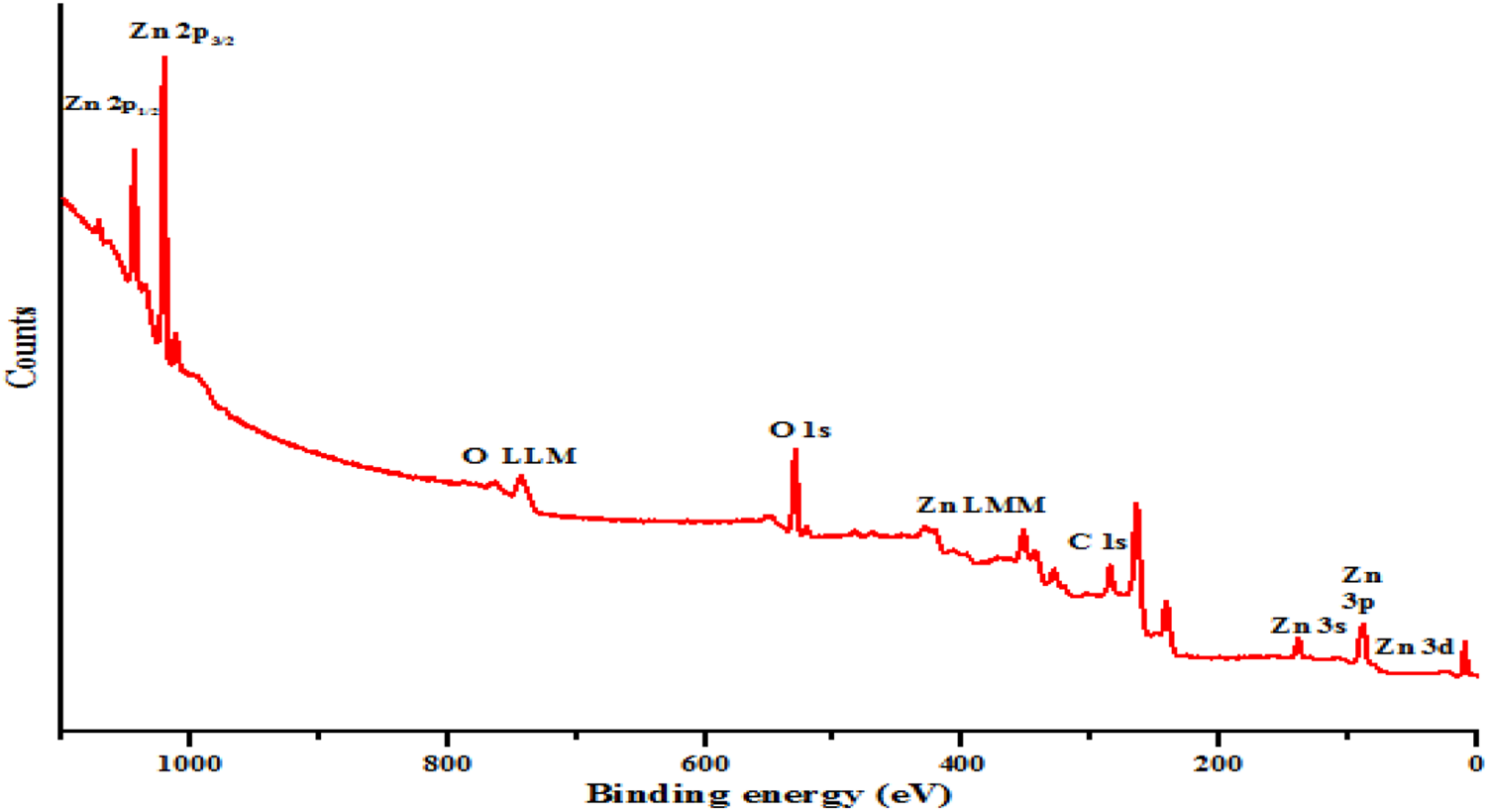
XPS general survey spectra of ZnO nanoparticles.

**Figure 9:**
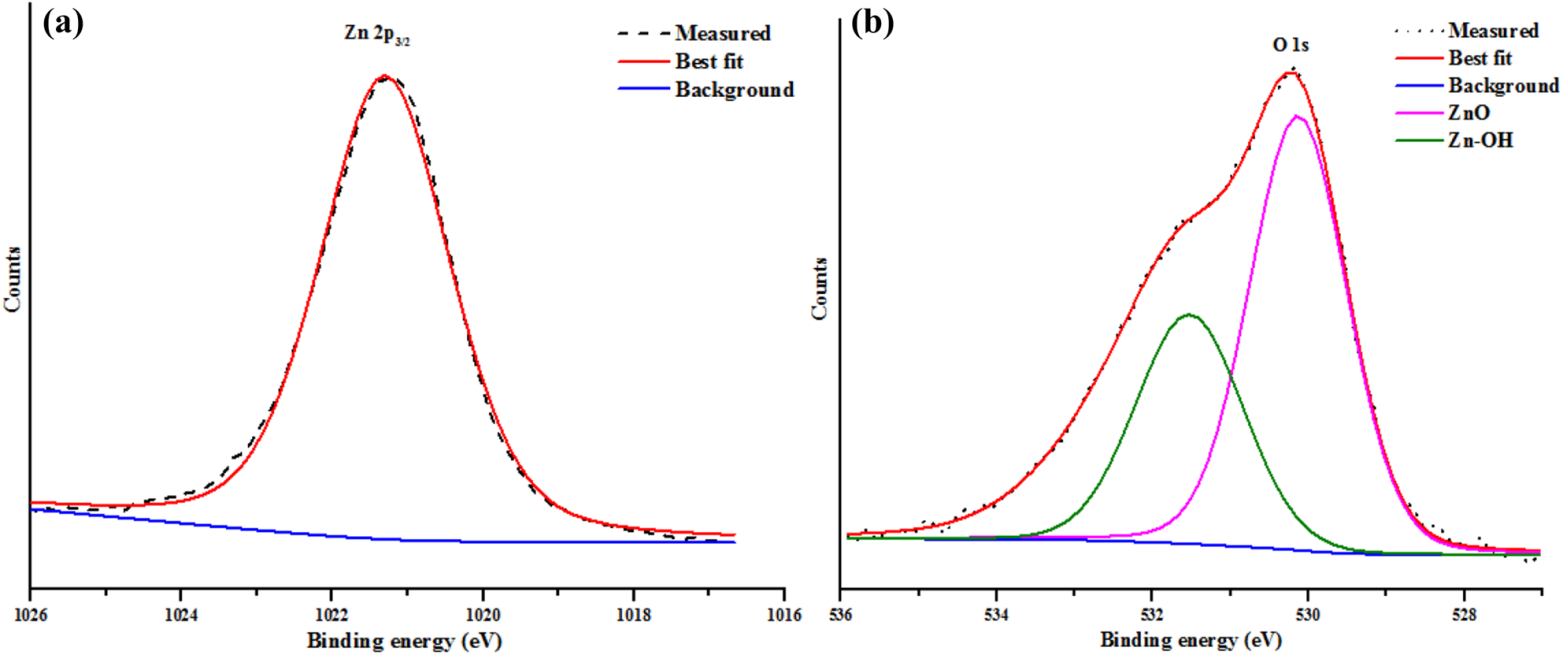
XPS profiles of ZnO nanoparticles for (**a**) Zn and (**b**) O 1*s*.

The surface chemical states of Zn and O in the prepared ZnO nanoparticles and kaolin/ZnO nanocomposites were examined using XPS and the results are shown in Figs. 8 and 9 respectively. Figure 8 shows the general survey of XPS survey of prepared ZnO nanoparticles and revealed the presence of strong signals of Zn (2*p*), O LLM, O (1*s*), Zn (LMM), C (1*s*), Zn (3*s*), Zn (3*p*) and Zn (3*d*) at different binding energies. It is should be mentioned that below the binding energy of 200 eV, Zn exists in the following orbital types (Zn 3*s*, 3*p* and 3*d*). While two peaks of Zn found in the binding energies of 1,021 and 1,045 eV ascribed to spin–orbit coupling corresponds to Zn 2*p*_3/2_ and Zn 2*p*_1/2_ and further confirmed the existence of Zn in ZnO environment in the valence states of + 2. The energy differences between the doublet peaks (Zn 2*p*_3/2_ and Zn 2*p*_1/2_ orbitals) is 24 eV, which is similar to that of typical ZnO nanoparticles. This is in good agreement with previous studies^27,30^. Figure 9a which represent the high-resolution XPS spectra of Zn (2*p*) core level region of ZnO nanoparticles revealed the presence of a sharp singlet peak at a binding energy of 1,021.30 eV. The singlet peak was attributed to the Zn 2*p*_3/2_ orbital and implies that the Zn element exists in the Zn^2+^ chemical state. The quantitative analysis shows that the atomic percentage of Zn and O are 79.24 and 18.90%, respectively, which agreed with the theoretical values of Zn and O of ZnO.

As shown in Fig. 9b, the binding energy of the O (1*s*) region for the ZnO nanoparticles revealed the presence of two sharp and intense peaks at around 529.5 eV and 532.4 eV, indicating lattice oxygen O^2−^ ions in the Zn–O bonding of the zincite phase of ZnO. While the peak detected at 532.4 eV is assigned to the Zn–OH group adsorbed on the surface of the ZnO nanoparticles. This consistent with strong FTIR signal shown in Fig. 3 that confirmed the presence of O–H in kaolin/ZnO composites This further corroborates previous reports on ZnO nanoparticles^31^.

**Figure 10:**
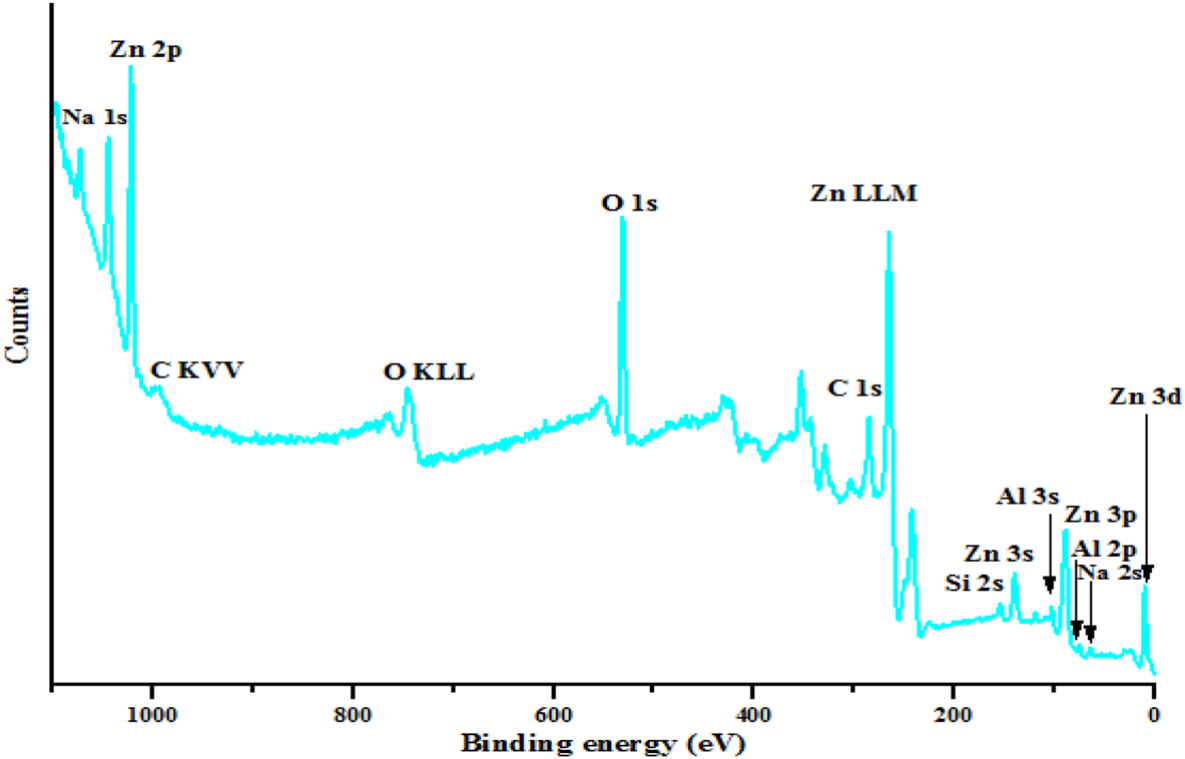
XPS general survey spectra of ZnO/kaolin nanocomposites.

**Figure 11:**
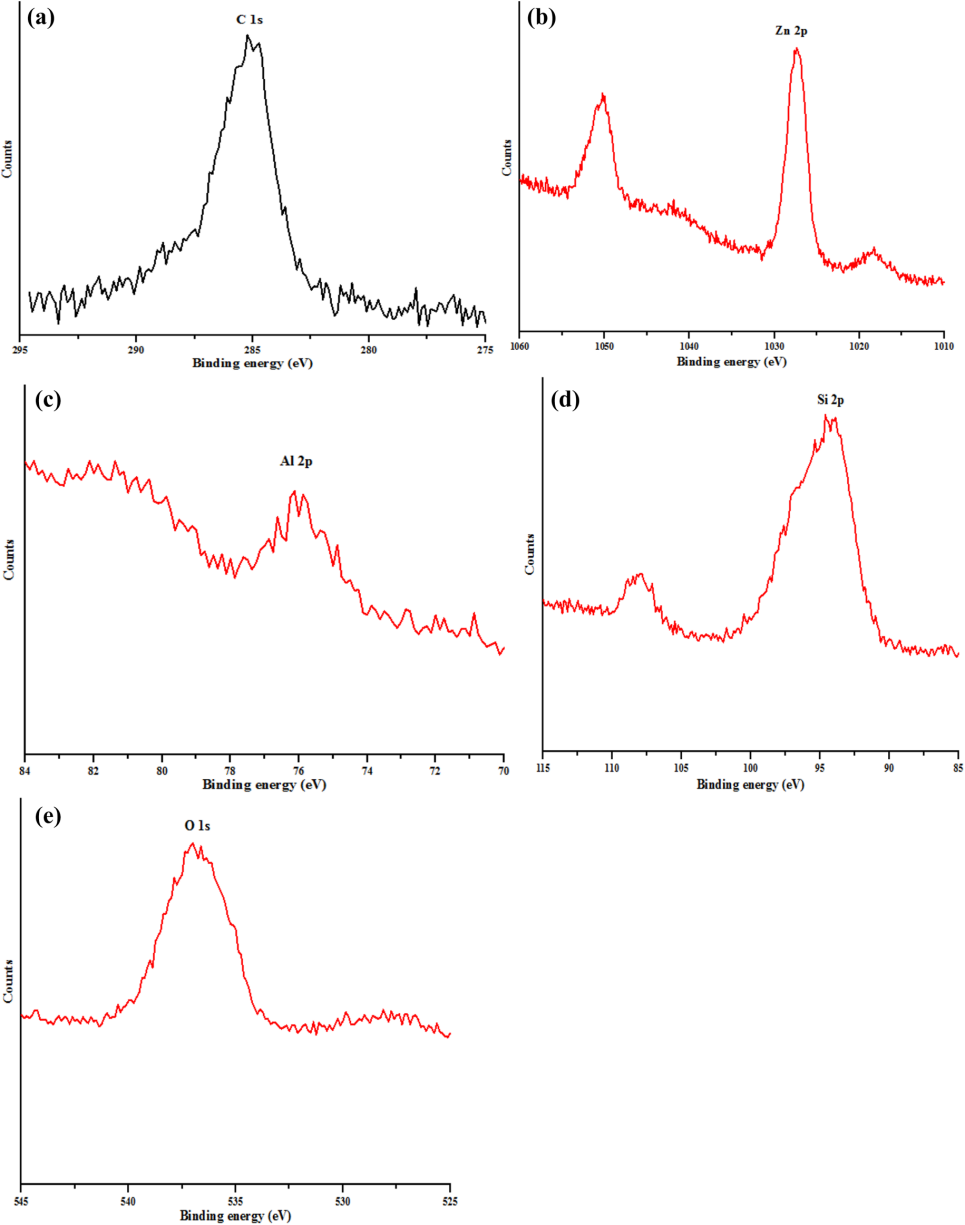
High resolution of XPS spectra of (**a**) C 1*s*, (**b**) Zn 2*p*, (**c**) Al 2*p*, (**d**) Si 2*p* and (*e*) O 1*s* for the synthesized ZnO/kaolin nanocomposites.

The XPS general survey of kaolin/ZnO nanocomposites displays in Fig. 10 revealed the following dominant elements and orbital types (Na 1*s*, Zn 2*p*, Zn 3*s*, Zn 3*p*, Zn 3*d*, Na 2*s*, Si 2*s*, C 1*s*, Al 2*s* and Al 3*p*) at different binding energies. The elements detected are key constituted in the samples except for Na which originated from NaOH used as a precipitating agent. The detected carbon (C 1*s*) at the binding energy of 286.4 eV in the deconvoluted XPS spectra shown in Fig. 11a can be from two sources either (1) carbon adsorbed on the surface during the exposure of the samples to ambient temperature or (2) zinc acetate dihydrate precursor used for the synthesis of ZnO nanoparticles^31^. Besides, deconvoluted XPS spectra of Zn core electrons presented in Fig. 11b revealed the presence of two strong peaks at binding energies of 1,027.9 eV and 1,050.9 eV respectively compared to one peak observed for Zn in ZnO (Fig. 9a) at 1,021.3 eV. The strong doublet peaks found binding energies of at 1,027.9 eV and 1,050.9 eV were assigned to Zn 2*p*_3/2_ and Zn 2*p*_1/2_ earlier observed in Fig. 9a. These values are in agreement with the binding energies of Zn^2+^ ion previously reported^32,33^. The shift in the binding energy of Zn to higher values in kaolin/ZnO relative to Zn in ZnO alone further suggest substitutional effects of Si or Al in kaolin with oxygen in ZnO, thereby creating surface defect and oxygen vacancies. Very importantly, despite the differences in the binding energies of Zn in kaolin/ZnO composites and Zn in ZnO nanoparticles, Zn (Zn 2*p*_3/2_ and Zn 2*p*_1/2_) still exists in the chemical states of + 2. This is because the energy differences between 1,050.9 and 1,027.9 eV, for Zn in kaolin/ZnO composites is 23 eV, which means immobilization of kaolin onto ZnO lattice layer did not change its oxidation state as well ss zincite phase of ZnO nanoparticles.

The deconvoluted XPS spectra of Al 2*p* and Si 2*p* shown in displayed in Fig. 11c, d respectively. The Al and Si originated from the kaolin and base on the difference in the ionic radius, Zn (139 pm) and O (152 pm) could diffuse into the Si-Al framework with the ionic radius of 210 pm and 184 pm respectively. This diffusion mechanism of Zn onto the kaolin was responsible for the morphological transformation of hexagonal-shaped kaolin to spherical particles. The XPS result further corroborates the HRTEM result earlier shown in Fig. 4. As such, Al and Si will capture oxygen in competition with Zn and result in more oxygen vacancies.

Unlike O 1*s* spectra in ZnO alone with two strong peaks, XPS deconvoluted spectra of O 1*s* shown in Fig. 11e have only one broad peak at a binding energy of 537.5 eV, which correspond to chemisorbed oxygen and further confirmed the existence of surface defects in kaolin/ZnO composites (Fig. 10).

### Adsorption studies

#### Effect of contact time

**Figure 12:**
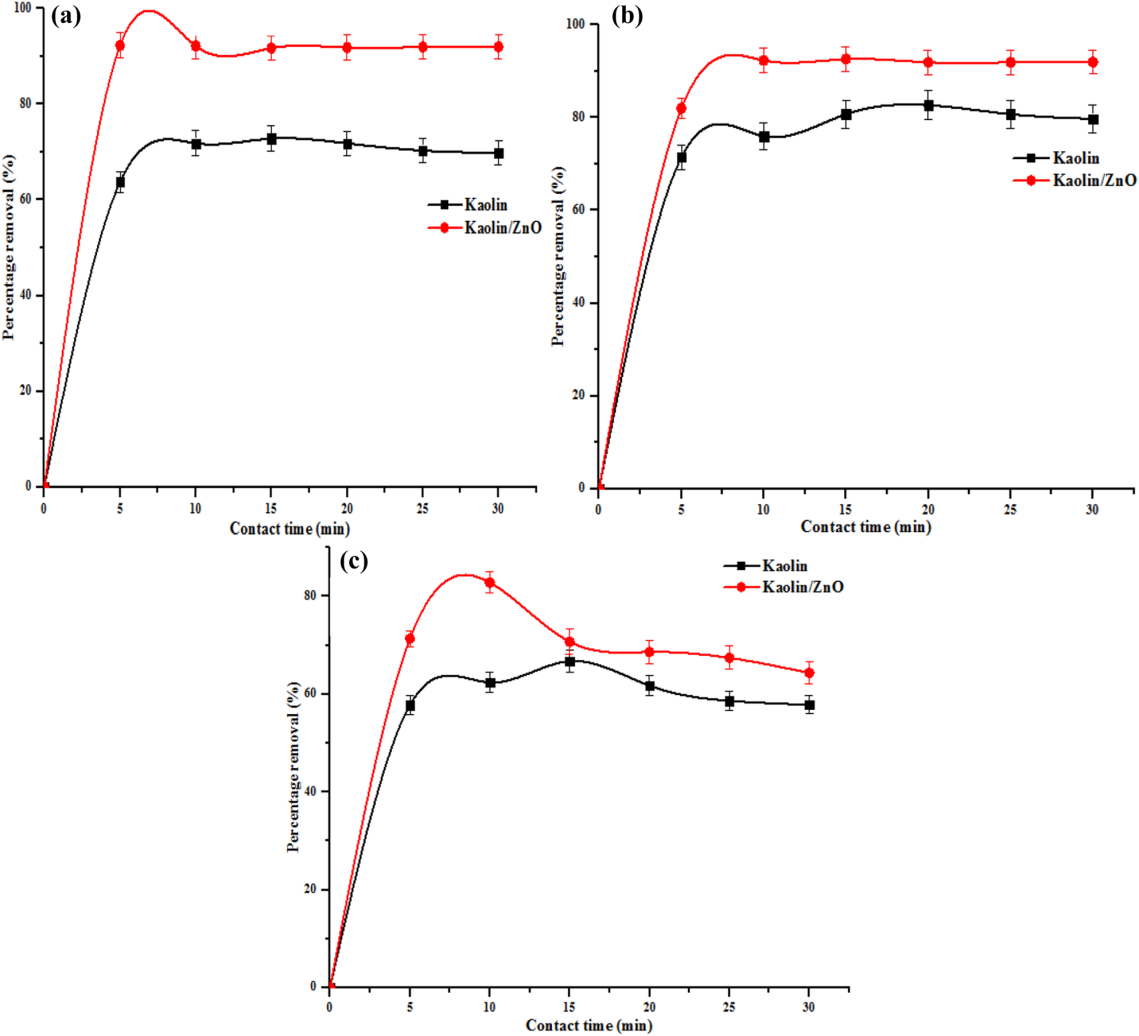
Effect of contact time on (**a**) COD (**b**) BOD and (**c**) chloride removal onto kaolin and kaolin/ZnO (adsorbent dose 0.2 g, agitation speed 150 rpm, temperature 29 °C and pH 5.84). Errors bars signify means ± standard errors from the mean of duplicate experiments.

**Figure 13:**
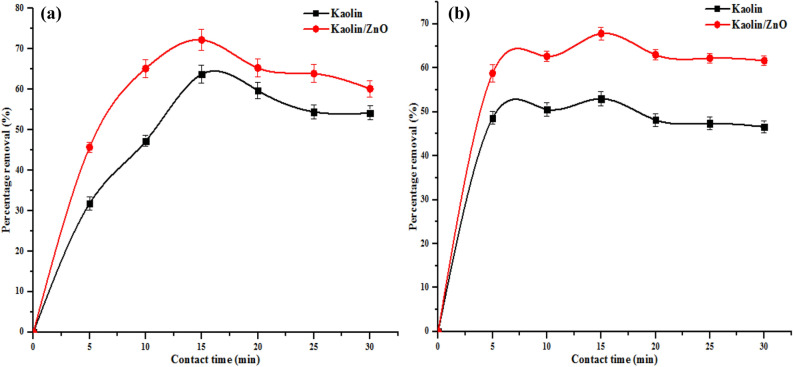
Effect of contact time on (**a**) Cr and (**b**) Fe ions removal onto kaolin and kaolin/ZnO (adsorbent dose 0.2 g, agitation speed 150 rpm, temperature 29 °C and pH 5.84). Errors bars signify means ± standard errors from the mean of duplicate experiments.

**Figure 14:**
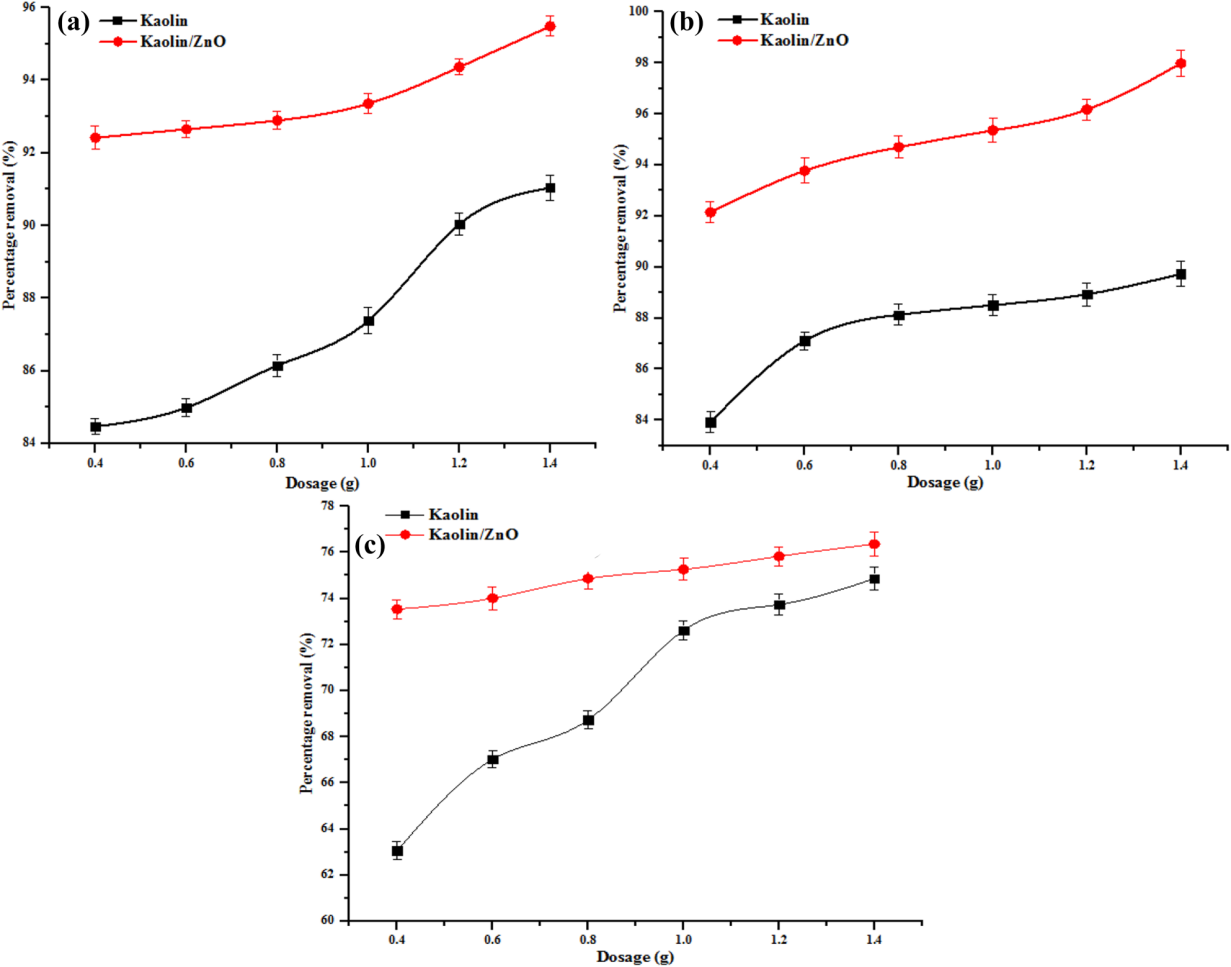
Effect of dosage on (**a**) COD, (**b**) BOD and (**c**) chloride removal onto kaolin and kaolin/ZnO (agitation speed 150 rpm, contact time, 15 min, temperature 29 °C and pH 5.84). Errors bars signify means ± standard errors from the mean of duplicate experiments.

The influence of contact time on the sequestration of pollutants on the adsorbent is paramount to determine when equilibrium adsorption is attained. Figures 12 and 13 show the effect of contact time on the percentage removal of COD, BOD, chloride and heavy metals (Cr and Fe) ions utilizing kaolin and kaolin/ZnO nanocomposites. As shown in Fig. 13a–c, amount of COD, BOD and chloride removed increase with increasing contact time up till 10 min irrespective of the adsorbents. Beyond 10 min, there was no significant improvement in the level of reduction of COD and BOD instead of the curve and the value remain constant. On the other hand, for chloride (Fig. 13c), after 10 min, the amount of chloride sorbed by the two materials reduced. Thus, 15 min was considered as the optimum equilibrium time for the two adsorbents because the additional increase in the time did not increase the uptake rates of the pollutants due to the deposition of the pollutants on the surface of the adsorbents. The fast removal rate during the first 10 min was ascribed to the availability of binding sites on the surfaces of kaolin and kaolin/ZnO nanocomposites. The maximum COD, BOD and chlorides removed by kaolin/ZnO composites under the applied conditions were 93%, 96% and 91%. While the removal efficiency of the indicator parameters by kaolin alone were: 65%, 67% and 62%, respectively. Based on the results obtained, the removal of pollutants was rapid in the early stages and the reduction in scavenging of the metal ions at the later stage of the adsorption process was due to desorption and over-saturation or reduction of available active adsorption sites. It was noticed that at every contact time, kaolin/ZnO composites removed more of COD, BOD and chlorides than kaolin alone due to higher surface area (31.8 m^2^/g) of the former than the latter (kaolin 17 m^2^/g). A similar trend was observed in Fig. 14a, b on the removal of Fe and Cr ions from tannery wastewater by the two materials. It was found that the adsorbents (kaolin/ZnO composites, kaolin) removed more of Cr (75%, 60%) than Fe (69%, 50%) ions under the applied conditions. The mesoporosity nature of the kaolin also contributed to the improved and better performance of kaolin/ZnO composites than kaolin alone. The faster rate of adsorption of Cr ion, when compared to Fe ion, may be attributed to the fact that metal ion with smaller ionic radii diffuses faster than ion with larger ionic radii. According to literature, the ionic radii of Cr^6+^ ion (0.052 nm) is smaller than Fe^3+^ ion (0.0645 nm) which makes it to diffuse faster to the pores of the adsorbents as a result of its smaller size. A similar trend was observed by Ogbu et al.^34^ and El-Naggar et al.^35^ who independently utilized 50%:50% mixture of kaolinite and sawdust and kaolinite/smectite composites to remove (Cd and Pb ions) and Pb ion from simulated wastewater after 45 min and 120 min respectively. Singh et al.^28^ reported and achieved 100% removal efficiency for Pb^2+^, Cd^2+^, As^3+^, and Hg^2+^ using Fe_3_O_4_/ZnO composites from simulated wastewater after 24 h. Zolfaghari et al.^18^ achieved 87% Pb removal rate within 120 min using ZnO/coated nanoporous carbon. The differences in the adsorptive performance of the materials and contact time can be linked to their surface areas and functional groups. While Singh et al.^28^ reported surface area of 43 m^2^/g for Fe_3_O_4_/ZnO composites, in this study surface area of 31.8 m^2^/g was obtained for kaolin/ZnO composites. It is important to mention that 100% removal efficiency of the target pollutants was not achieved under the influence of contact time and can be due to the following. (1) presence of several scavengers or other interfering metal ions in the tannery wastewater which also competes for active surface sites compared to the work of Singh et al.^28^ who reported 100% removal rate. Singh et al. used simulated wastewater that did not contain scavengers such as sulphates, carbonates, nitrates, phosphates and others. (2) the quantity of adsorbent used was small and did not cover the entire surface of wastewater and as such no proper interaction with the adsorbate. From the results, the efficiency of an adsorbent depends on its adsorption capacity and how fast metal ions can be removed from wastewater. In this regard, kaolin/ZnO nanocomposite was found to be more effective in removing pollutants compared to pure kaolin. Thus, the potential of immobilized ZnO on kaolin as nanocomposites adsorbent for the removal of metal ions from wastewater is demonstrated.

#### Effect of adsorbent dosage

**Figure 15:**
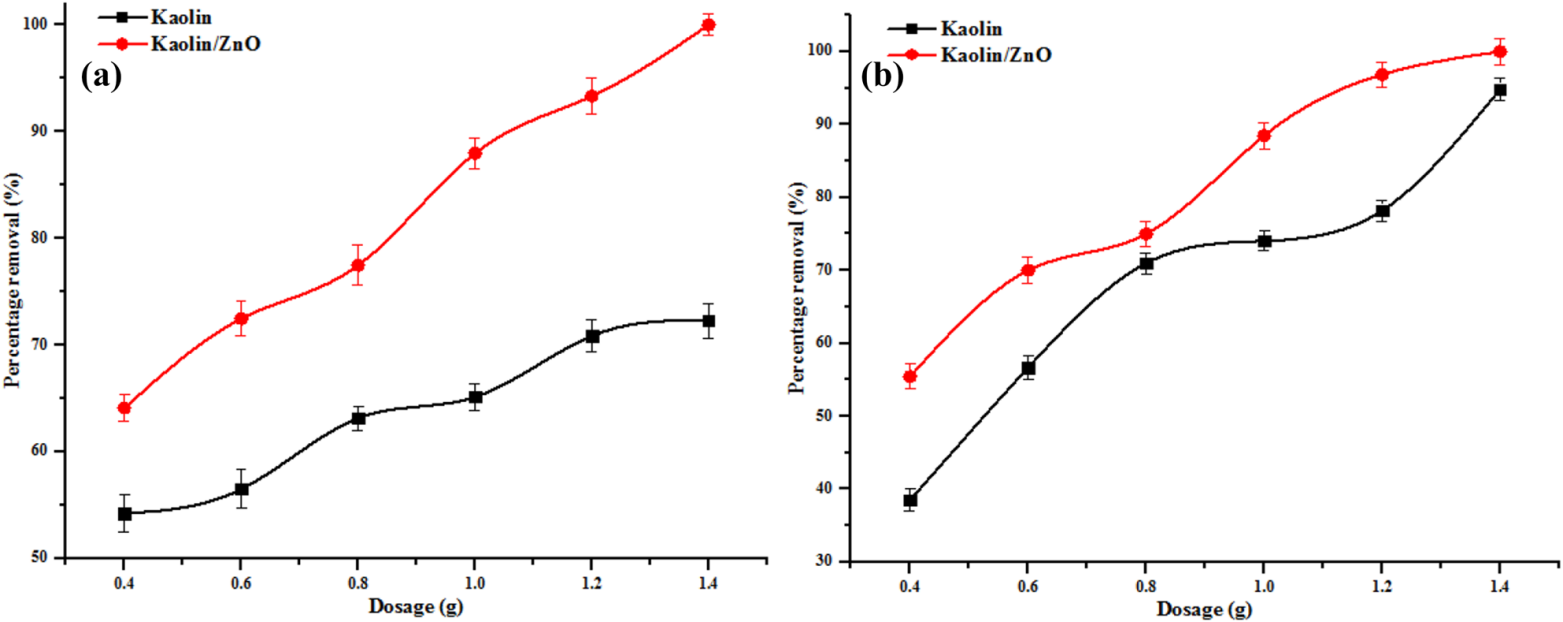
Effect of dosage on (**a**) Cr and (**b**) Fe ions removal onto kaolin and kaolin/ZnO (agitation speed 150 rpm, contact time, 15 min, temperature 29 °C and pH 5.84). Errors bars signify means ± standard errors from the mean of duplicate experiments.

The scavenging of metal ions depends on the nature and amount of the adsorbent. The effect of adsorbent dosage on removal of COD, BOD, chloride and heavy metals (Cr^6+^ and Fe^3+^) are shown in Figs. 14 and 15, respectively. The removal efficiency of metal ions, COD, BOD and chloride increased with increase in adsorbent dosage from 0.2 to 1.4 g which implies that there was an increase in adsorption of pollutants for kaolin and kaolin/ZnO nanocomposites. The simultaneous increase in metal ion uptake and other indicator parameters (COD, BOD and chloride) with increase dosage can be explained in terms of surface functionality, greater availability of more active or exchangeable sites and increased surface area of the adsorbents. The mechanism of removal of Cr(VI) and Fe(III) ions can be explained as follow: kaolin and Kaolin/ZnO composites possess negative charges and through electrostatic interactions, the positively charged species like Cr(VI) and Fe(III) ions, can be removed from tannery wastewater. Similar explanation hold for COD, BOD and chloride. The polar and porous network structure of ZnO nanoparticles which provides more active sites for the adsorption of metal ions may also be responsible for greater removal efficiency by kaolin/ZnO composites than kaolin alone. On the other hand, the high electronegativity values of Fe(III) (1.83) than that of Cr(VI) (1.66) did not suggest removal of more Fe(III) than Cr(VI). Instead more of Cr(VI) was removed by the two adsorbents than Fe(III). This shows that the mechanism of heavy metal ions adsorption from aqueous solution by the adsorbent did not depend on the electronegativity parameter but rather on surface areas and surface functionality^17^. The excellent 100% and 98% removal efficiency achieved for Cr(VI) and (Fe(II) at a higher dose of kaolin/ZnO composites compared to kaolin can be linked-to existence of synergetic effects between the two materials as evidence in the number of functional groups shown in Fig. 3. Also, at every dose of the adsorbents, kaolin/ZnO nanocomposites adsorbed metal ions, COD, BOD and chloride at a higher rate than kaolin due to an increase in adsorption surface area and a decrease in diffusion path length leading to less aggregation of adsorption sites. The adsorption trend of metal ions uptake onto the adsorbents was in the order kaolin/ZnO nanocomposites > kaolin due to ZnO network structures which provides extra active binding sites on the former than the latter. The trend in this study corroborates the previous studies on the removal of Cr(VI) and other pollutants^9^. The maximum uptake of some pollutants by the adsorbents was compared with other adsorbents in literature as shown in Table 1. Taking into account that is cumbersome to compare the adsorption capacities of the tannery wastewater with different adsorbents, since the tannery wastewater depends on its constituents. The prepared and synthesized kaolin/ZnO nanocomposites in this study show its suitability for use as an adsorbent for the removal of the metal ions and other pollutants from wastewater.

**Table 1:** Comparison of the percentage removal of pollutant using different nanoadsorbents.

Nanoadsorbent	Experimental condition	Maximum percentage removal (%)	Pollutant	References
ZnO/montmorillonite	Contact time (240 min), pH (7), dosage (0.0–0.15 g)	88	Phenol	Ye et al.^36^
ZnO/montmorillonite	Contact time (160 min), dosage (250 mg), pH (6.7)	74	Textile azo dye	Boutra and Trari^37^
ZnO/bentonite	Contact time (70 min), pH (4), dosage (0.4–1.4 g/L)	96.8	Safranine	Sonawane et al.^20^
Mg(OH)_2_/bentonite	Contact time (3 h), pH (6.5), dosage (1–5 g/L)	70	Anionic reactive dye	Chinoune et al.^38^
Na-activated bentonite	Contact time (40 min), dosage (0.125–0.5 g), pH (7)	95 and 85	Pb^2+^ and Cd^2+^	Taha et al.^39^
Polyacrylic acid/organobentonite	Contact time (30 min), dosage (0.02–0.3 g)	99.6	Pb^2+^	Rafiei et al.^40^
ZnO/montmorillonite	Contact time (2 h), pH (4), doage (0.02–0.1 g)	98	Pb^2+^	Sani et al.^4^
Wood activated carbon/zerovalent iron	Contact time (30 min), pH (6), dosage (10–150 mg), temperature (25 °C)	97.3	Pb^2+^	Dada et al.^41^
ZnO/kaolin	Contact time (15 min), pH (5.84), dosage (0.4–1.4 g), temperature (29 °C)	100	Cr^6+^	Present study
ZnO/kaolin	Contact time (15 min), pH (5.84), dosage (0.4–1.4 g), temperature (29 °C)	99.8	Fe^3+^	Present study

#### Effect of temperature

**Figure 16:**
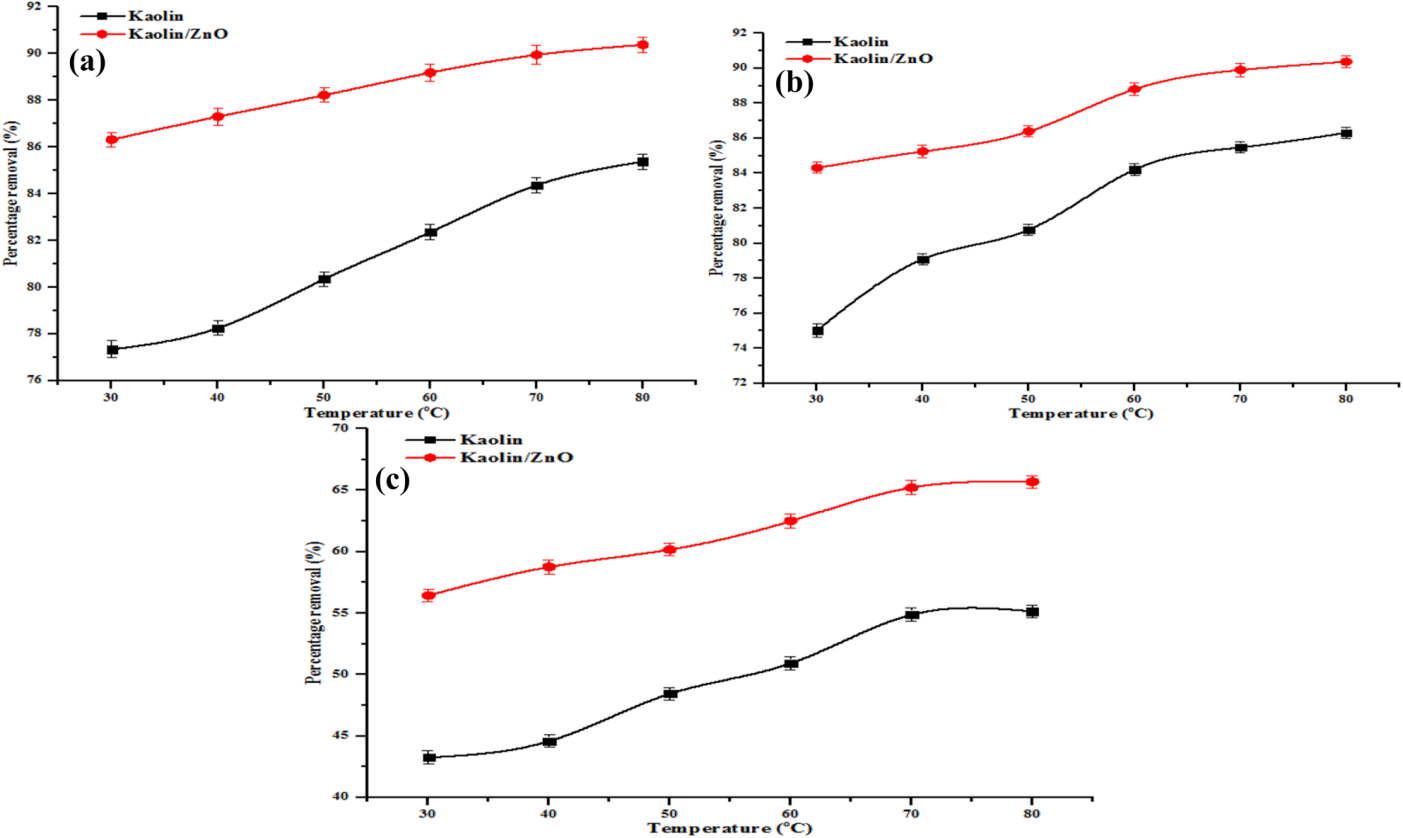
Effect of temperature on (**a**) COD, (**b**) BOD and (**c**) chloride removal onto kaolin and kaolin/ZnO (adsorbent dosage, 0.2 g, contact time, 15 min, agitation speed 150 rpm and pH 5.84). Errors bars signify means ± standard errors from the mean of duplicate experiments.

**Figure 17:**
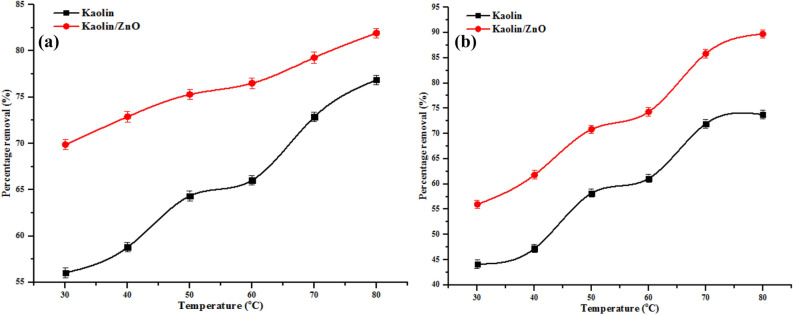
Effect of temperature on (**a**) Cr and (**b**) Fe ion removal onto kaolin and kaolin/ZnO (adsorbent dosage, 0.2 g, contact time, 15 min, agitation speed 150 rpm and pH 5.84). Errors bars signify means ± standard errors from the mean of duplicate experiments.

The effect of temperature on the adsorption of COD, BOD, chloride and heavy metals (Cr(VI)and Fe(III)) onto kaolin and kaolin/ZnO samples were investigated at 30, 40, 50, 60, 70 and 80 °C as presented in Figs. 16 and 17. It can be noticed that the uptake of COD, BOD, chloride and metal ions (see Figs. 16, 17) increased with increase in temperature from 30 to 80 °C. The enhanced in the removal efficiency of COD, BOD, chloride, Cr(VI) and Fe(III) as a function of temperature may be ascribed to increased mobility and diffusion of the ionic species and other pollutants onto the pores of the adsorbents. This further suggests strong chemical interaction between the (metal ions and other pollutants) with the surface functional groups on the adsorbents. The trend obtained is evidence of a direct relationship between percentage removal efficiency and temperature. This implies that the sorption of the pollutants onto kaolin and kaolin/ZnO adsorbents is endothermic, and can equally be referred to as heterogeneous and reversible process. Comparatively, it was found that the rate of adsorption of metal ions and other pollutants followed the order kaolin/ZnO composites > kaolin. This result clearly illustrated that high surface coverage, high surface area, creation of more active and reactive sites as a result of anchoring of kaolin onto ZnO nanoparticles improved the adsorption of the nanocomposites in this study. The effect of temperature on the adsorption of COD, BOD, chloride, Cr(VI) and Fe(III) leading to estimation of Gibbs free energy, entropy and enthalpy of the reaction is shown in Table 3.

### Adsorption isotherm studies

#### Jovanovic isotherm

Jovanovic model is similar to the Langmuir model and describes the surface binding vibration of the adsorbate or the possibility of a mechanical constant between the sorbate and sorbent. This model is shown using Eq. (3).

3 $$ {\text{lnq}}_{{\text{e}}} = {\text{lnq}}_{{{\text{e}}\left( {\max} \right)}} - {\text{K}}_{{\text{j}}} {\text{C}}_{{\text{e}}} $$

where ${\text{K}}_{{\text{j}}}$ is a Jovanovic constant and ${\text{q}}_{{{\text{e}}\left( {\max} \right)}}$ is the maximum adsorption capacity pollutants uptake. The relationship between ${\text{lnq}}_{{\text{e}}}$ and ${\text{C}}_{{\text{e}}}$ for the removal of COD, BOD, chloride and metal ions give straight lines. The results of the slope (${\text{K}}_{{\text{j}}}$), ${\text{q}}_{{\text{e}}}$, and correlation coefficient (R^2^) are given in Table 2. The calculated constants for the Jovanovic isotherm model presented in Table 2 show a slight increase in correlation coefficient values (R^2^ > 0.99). This indicates that monolayer adsorption is the major mechanism for the removal of pollutants using kaolin and kaolin/ZnO nanocomposites.

**Table 2:** Jovanovic, Halsey, Flory–Huggins and Redlich–Peterson isotherm parameter for adsorption of some parameters in tannery wastewater onto kaolin and kaolin/ZnO (KZ).

Parameter	Sample	Jovanovic			Halsey			Flory-H			Redlich-P		
		${q}_{max}$	${K}_{j}$	${R}^{2}$	${n}_{H}$	${I}_{n}{K}_{H}$	${R}^{2}$	$n$	${K}_{FH}$	${R}^{2}$	$\beta$	$A$	${R}^{2}$
Chloride	Kaolin	1694.21	2.046	0.99971	0.917	14.712	0.99501	− 1.023	3.218 × 10^–5^	0.99501	2.023	3.449 × 10^6^	0.99871
	KZ	1715.4	2.128	0.99982	1.592	18.861	099,629	− 0.628	4.938 × 10^–5^	0.99629	1.628	1.393 × 10^5^	0.99942
COD	Kaolin	369.814	5.568	0.99979	4.474	31.756	0.99434	− 0.224	2.800 × 10^–4^	0.99434	1.224	1.210 × 10^3^	0.99981
	KZ	380.615	5.672	0.99990	7.689	50.507	0.99625	− 0.130	3.350 × 10^–4^	0.99625	1.130	7.126 × 10^2^	0.99985
BOD	Kaolin	125.015	1.690	0.99949	4.360	24.885	0.98658	− 0.229	8.793 × 10^–4^	0.98658	1.229	3.011 × 10^2^	0.99853
	KZ	125.965	1.810	0.99992	7.176	37.563	0.99509	− 0.141	1.040 × 10^–3^	0.99509	1.141	2.020 × 10^2^	0.99992
Iron	Kaolin	90.418	0.709	0.99085	0.642	0.170	0.98315	− 1.558	9.865 × 10^–2^	0.97315	2.558	1.303	0.98990
	KZ	95.493	0.847	0.99819	4.072	2.290	0.98487	− 0.245	2.665 × 10^–1^	0.82487	1.245	1.754	0.99214
Chromium	Kaolin	106.915	0.146	0.99960	2.129	4.258	0.98028	− 0.470	4.689 × 10^–2^	0.98028	1.470	17.46	0.99796
	KZ	117.253	0.157	0.99990	3.234	8.869	0.98602	− 0.309	5.856 × 10^–2^	0.98602	1.309	15.520	0.99921

*KZ* kaolin/ZnO nanocomposites.

#### Halsey isotherm

Halsey adsorption isotherm models can be used for the discussion of the multilayer adsorption at a relatively large distance from the surface. The fitting of the results is responsible for the porosity nature of the nanoadsorbent. Halsey adsorption isotherm model is given in Eq. (4).

4 $$ {\text{q}}_{{\text{e}}} = \frac{1}{{{\text{n}}_{{\text{H}}} }}{\text{I}}_{{\text{n}}} {\text{K}}_{{\text{H}}} - \frac{1}{{{\text{n}}_{{\text{H}}} }}{\text{lnC}}_{{\text{e}}} $$

where ${\text{n}}_{{\text{H}}}$ and ${\text{I}}_{{\text{n}}} {\text{K}}_{{\text{H}}}$ Halsey isotherm constants. The plots of ${\text{q}}_{{\text{e}}}$ against ${\text{lnC}}_{{\text{e}}}$ for the removal of COD, BOD, chloride and metal ions onto kaolin and kaolin/ZnO nanocomposites describe Halsey model isotherm. The isotherm constant values of the parameter model were presented in Table 2. It can be seen from Table 2 that the R^2^ values are > 0.98 but less than R^2^ values of Jovanovic isotherm. The experimental data showed that there is a lower likelihood of multilayer adsorption on the nanoadsorbents. The low correlation coefficient values of the Halsey adsorption isotherm indicate that it is not a good fit for the experimental data for pollutant removal using these adsorbents.

#### Flory–Huggins isotherm

The Flory–Huggins isotherm model accounts for the degree of surface coverage characteristics of the adsorbate onto the adsorbent. This is the simplest model allowing the feasibility and spontaneous nature of an adsorption process. Flory–Huggins adsorption isotherm model is given in Eq. (5).

5 $$ {\ln}\frac{{\uptheta }}{{{\text{C}}_{{\text{o}}} }} = {\text{lnK}}_{{{\text{FH}}}} + {\text{nln}}\left( {1 - {\uptheta }} \right) $$

6 $$ {\uptheta } = \left( {1 - \frac{{{\text{C}}_{{\text{e}}} }}{{{\text{C}}_{{\text{o}}} }}} \right) $$

where ${\text{K}}_{{{\text{FH}}}}$ is Flory–Huggins constant and n is the numerical size parameter of pollutants occupying sorption sites. The ${\text{K}}_{{{\text{FH}}}}$ positive values obtained from the intercepts represent Flory–Huggins isotherm for these adsorbents (as seen in Table 2), indicating that the Flory–Huggins isotherm model is applicable for modelling the adsorption of pollutants onto the adsorbents. This shows that the formation of multilayer of adsorbate molecules on the adsorbent surfaces can be predicted. Thus, the value of n less than 1 shows that the adsorbate molecule will occupy more than one active site.

#### Redlich–Peterson isotherm

The Redlich–Peterson isotherm model incorporated the hybrid features of Langmuir and Freundlich isotherms. In this present study, this model describes the mono and multilayer adsorption of pollutants from aqueous system using kaolin and kaolin/ZnO nanocomposites. The linear R–P isotherm is given in Eq. (7).

7 $$ {\ln}\frac{{{\text{C}}_{{\text{e}}} }}{{{\text{q}}_{{\text{e}}} }} =\upbeta {\text{lnC}}_{{\text{e}}} - {\text{lnA}} $$

where β and A are the Redlich–Peterson constants. The plots of ${\ln}\frac{{{\text{C}}_{{\text{e}}} }}{{{\text{q}}_{{\text{e}}} }}$ against ${\text{lnC}}_{{\text{e}}}$ for the removal of COD, BOD, chloride and metal ions from tannery wastewater describe the Redlich-Peterson isotherm. From the Redlich-Peterson plot, the slope and the intercept were determined. The values of R^2^, β and A are presented in Table 2 with values of β found to be above 1. From the three-parameter model (R–P) equation in this present work, it showed that Langmuir isotherm was more favoured than the Freundlich model. Hence, the highest R^2^ values for the isotherm models suggest that it is the most suitable isotherm model that models the equilibrium adsorption of COD, BOD, chloride and metal ions (Cr and Fe) by adsorbents. Generally, adsorption of these pollutants onto kaolin and kaolin/ZnO nanocomposites is best described by the isotherms model. In this study, the isotherm models fit the equilibrium data in the following order: Jovanovic > Redlich–Peterson > Flory–Huggins > Halsey.

### Thermodynamic studies

The thermodynamic parameters values were estimated to evaluate the feasibility of adsorption of metal ions onto the adsorbents. The distribution coefficient, ${\text{K}}_{{\text{D}}}$, was calculated from Eq. (8)

8 $$ {\text{K}}_{{\text{D}}} = \frac{{{\text{q}}_{{\text{e}}} }}{{{\text{C}}_{{\text{e}}} }} $$

The Gibbs free energy, enthalpy and entropy at different temperatures were calculated from the Vant Hoff’s plot using Eq. (9).

9 $$ {\text{lnK}}_{{\text{D}}} = \frac{{\Delta {\text{S}}}}{{\text{R}}} - \frac{{\Delta {\text{H}}}}{{{\text{RT}}}} $$

where R is the universal gas constant (8.314 J/molK), T is the absolute temperature (K), $\Delta {\text{S}}$ is the change in entropy (J/molK) and $\Delta {\text{H}}$ is the change in enthalpy (J/mol). The change in free Gibbs energy ($\Delta {\text{G}}$) (kJ/mol) values were calculated from Eq. (10).

10 $$ \Delta {\text{G}} = \Delta {\text{H}} - {\text{T}}\Delta {\text{S}} $$

The thermodynamic parameters are listed in Table 3 and it can be observed that the $\Delta {\text{H}}$ values were positive and further confirmed that the adsorption is an endothermic process. It can be noticed that the calculated $\Delta H$ was less than 40 kJ/mol for the removal of the pollutants by the two adsorbents. This is evidence of physisorption dominance over chemisorptions process of the adsorbates onto the adsorbents^42^.

**Table 3:** Thermodynamic parameters of some parameters in wastewater adsorption on kaolin and kaolin/ZnO at different temperature.

Parameter	Sample	$\Delta G (kJ/mol)$								
		${R}^{2}$	$\Delta H (kJ/mol)$	$\Delta S (J/molK)$	303 K	313 K	323 K	333 K	343 K	353 K
Chloride	Kaolin	0.96286	9.485	15.456	4.802	4.647	4.493	4.338	4.184	4.029
	KZ	0.97805	7.312	12.878	3.410	3.281	3.152	3.024	2.800	2.766
COD	Kaolin	0.8856	9.827	28.816	1.096	0.808	0.519	0.231	0.0569	− 0.345
	KZ	0.99558	7.317	26.064	− 0.580	− 0.841	− 1.102	− 1.362	− 1.623	− 1.884
BOD	Kaolin	0.973	13.47	40.48	1.205	0.800	0.395	0.00984	− 0.415	− 0.819
	KZ	0.95733	10.931	36.357	0.0852	− 0.449	− 0.812	− 1.176	− 1.540	− 1.903
Iron	Kaolin	0.90400	24.631	77.825	1.053	0.272	− 0.507	− 1.285	− 2.063	− 2.841
	KZ	0.91706	11.838	35.159	1.185	0.833	0.482	0.130	− 0.222	− 0.573
Chromium	Kaolin	0.90041	18.725	68.898	− 2.151	− 2.840	− 3.529	− 4.218	− 4.907	− 5.596
	KZ	0.97623	11.331	50.073	3.841	− 4.342	− 4.843	− 5.343	− 5.844	− 6.345

The positive values of $\Delta S$ suggested an increased in the degree of randomness or disorderliness at the adsorbate–adsorbent interface. The disorderliness may be ascribed to the increment in the translational entropy of the displaced water molecules relative to that lost by the pollutants during adsorption process^42^.

The negative and positive values of $\Delta {\text{G}}$ in the temperature ranges confirmed that the adsorption of metal ions onto adsorbents was spontaneous and non-spontaneous in nature. However, it was further confirmed that as the temperature increase, $\Delta {\text{G}}$ reduces (becoming negative), indicating that the adsorption processes became spontaneous and highly favoured at high temperature.

#### Adsorption kinetics and mechanism

The data from the effect of contact time was used to examine the adsorption mechanism and kinetics of metal ions onto the kaolin and kaolin/ZnO nanocomposites by fitting the experimental data to the intraparticle diffusion equation^43^ presented as follows:

11 $$ {\text{q}}_{{\text{t}}} = {\text{k}}_{{\text{i}}} {\text{t}}^{1/2} + {\text{I}} $$

where ${\text{q}}_{{\text{t}}}$ is the amount of metal ions loaded onto the adsorbent at time t (mg/g), ${\text{k}}_{{\text{i}}}$ is the intra-particle diffusion (mg/g), t is the contact time (min) and I is the intercept. The intra-particle diffusion model parameters were calculated from the plot of ${\text{q}}_{{\text{t}}}$ against t at various contact time. The linear profile of the plots of the COD, BOD, chloride and metal ions did not pass through the origin. This is an indication that some degree of boundary layer controlled the removal of metal ions, COD, BOD and chloride onto the adsorbents. The deviation from the origin may be due to mass transfer and adsorption at the initial and final stages, respectively. The intercept, I, values are greater than 0 as presented in Table 4, indicating that the intra-particle diffusion or internal diffusion was not only the rate-controlling step, but the film diffusion may also play an important role in the adsorption process. It was observed that there are two separate regions in which the first portion could be attributed to the bulk diffusion and the second portion to intra-particle diffusion for the removal of pollutants onto kaolin and kaolin/ZnO nanocomposites.

**Table 4:** Mechanism adsorption kinetic parameters of some physicochemical properties in tannery effluent.

Parameter	Sample	Boyd Model	Intra-Particle Model		
		${R}^{2}$	${K}_{id}$	$I$	${R}^{2}$
COD	Kaolin	0.81404	7.289	10.713	0.55510
	KZ	0.89255	12.065	13.406	0.57442
BOD	Kaolin	0.71006	3.022	6.166	0.66822
	KZ	0.89446	5.220	7.524	0.76476
Chloride	Kaolin	0.75083	6.0583	13.183	0.77301
	KZ	0.81174	7.649	18.791	0.86069
Iron	Kaolin	0.80660	1.8197	4.0709	0.80749
	KZ	0.96065	2.0149	5.0965	0.86209
Chromium	Kaolin	0.77958	2.0268	8.846	0.85207
	KZ	0.87150	3.0262	9.930	0.86996

The Boyd kinetic predicts the slow steps involved in the adsorption process. The Boyd kinetic equation^44^ is given as follows:

12 $$ {\text{F}} = 1 - \frac{6}{{\uppi }}{\exp}\left( { - {\text{B}}_{{\text{t}}} } \right) $$

13 $$ {\text{F}} = \frac{{{\text{q}}_{{\text{t}}} }}{{{\text{q}}_{{\text{e}}} }} $$

where ${\text{q}}_{{\text{t}}}$ and ${\text{q}}_{{\text{e}}}$ in (mg/g) are the amount of metal ion uptake at the time and maximum equilibrium uptake. F is the fraction of metal ion adsorbed at the time, t.

14 $$ {\text{B}}_{{\text{t}}} = - 0.4977 - {\ln}\left( {1 - {\text{F}}} \right) $$

**Figure 18:**
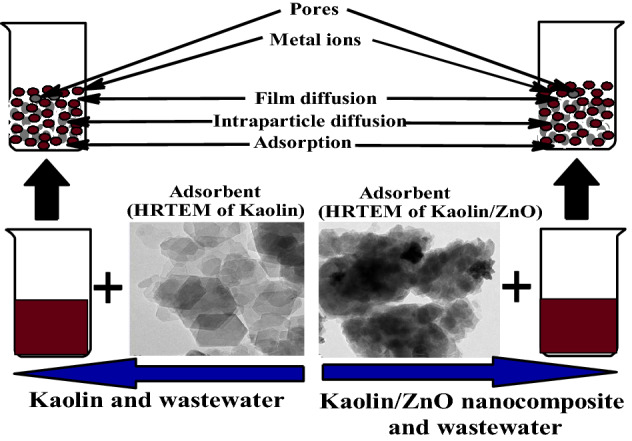
Steps in the adsorption mechanism.

${\text{B}}_{{\text{t}}}$ is the time constant and is a function of F from the linear plot of ${\text{B}}_{{\text{t}}}$ versus time, t. The Boyd plots proved that the linear plots did not pass through the origin and show that the adsorption process was governed by film or external diffusion. Generally, during the adsorption of pollutants onto the adsorbents, three adsorption mechanistic steps occur and are depicted in Fig. 18.

### Evaluation of the nanoadsorbent

Table 5 presents the comparison of the maximum adsorption capacities of different nanoadsorbents for the removal of Cr(VI) from wastewater solution. The high adsorption capacity (117.25 mg/g) showed that ZnO/kaolin nanocomposites was more effective nanoadsorbent for the removal of Cr(VI) from wastewater than previously reported nanoadsorbents. The lower contact time, high percentage removal, cost-effectiveness of the natural material used as support (kaolin), and non-toxic nature of the nanocomposites make this candidate promising and useful adsorbent. The demonstrated results from the study show the efficacy of ZnO/kaolin nanocomposites on industrial tannery wastewater and it can be employed to treat other industrial effluents. Therefore, it could be enlisted among effective nanoadsorbents for pollutants industrial wastewater treatment.

**Table 5:** Comparison of adsorption capacities of different adsorbents for sequestration of Cr(VI) ions.

Nanomaterial	Experimental condition	${Q}_{max}$ (mg/g)	Surface area (BET)	Isotherm	Kinetic	Particle size	References
ZnO/biochar	Contact time (14 h), dosage (0.2 g), pH (7), initial concentration (100 mg/L)	43.48	740.80	Langmuir		28.3	Yu et al.^22^
Guar gum/ZnO	Contact time (0.83 h), pH (7), dosage (1 g/L)	55.56		Langmuir	Pseudo-second-order, liquid film diffusion, intraparticle diffusion	Simulated	Khan et al.^42^
Fe_2_O_3_/ZnO	Contact time, pH, dosage, initial concentration	36.50	59.25	Langmuir	Lagergren and pseudo-second-order kinetic	Simulated	Olivera et al.^45^
ZnO	Contact time (90 min), pH (2), dosage (4 g/L), initial concentration (30 mg/L)	26.7		Langmuir	Pseudo-first-order, Pseudo-second-order	300	Pandey and Tripathi^46^
TiCN/ZnO	Contact time (50 min), pH (6), temperature (90 °C), dosage (0.1 g/L), initial concentration (500 mg/L)	0.34	3.96	Langmuir	Pseudo-first-order, Pseudo-second-order, intraparticle diffusion	Simulated	Rasaki et al.^47^
ZnO	Contact time (40 min), pH (3), initial concentration (100 mg/L), dosage (0.1 g)	0.00165		Langmuir	Pseudo-first-order, Pseudo-second-order	37	Kamath et al.^48^
ZnO/ZnS	Initial concentration (5–400 mg/L), contact time (24 h), pH 6, dosage (40 mg)	24.5	382.79	Langmuir	Pseudo-first-order, Pseudo-second-order, Elovich	33.4	Li et al.^49^
NiAl_2_O_4_/ZnO	Contact time (1 h), initial concentration (10–50 mg/L), dosage (0.05 g), pH (3.7)	4.27		Langmuir	Pseudo-first-order, Pseudo-second-order	10,000	Bouallouche et al.^50^
Polyamide/triazine/ZnO	Initial concentration (10 mg/L), pH (4),	101.01		Langmuir	Pseudo-first-order, Pseudo-second-order, Elovich, intra-particle diffusion	15–20	Dinari and Haghighi^51^
ZnO	Contact time (40 min), pH (8), initial concentration (1–5 mg/L)	9.38	15.75	Langmuir	Pseudo-first-order, pseudo-second-order	9.73	Kumar et al.^52^
Fe_3_O_4_/cetyltrimethylammonium bromide	Contact time (12 h), pH (4), dosage (12 mg/mL), concentration (20 mg/L)	18.50		Langmuir	Pseudo-first-order, Pseudo-second-order	10–20	Elfeky et al.^53^
VO_2_(B)	Contact time (360 min), dosage (100 mg), pH (7), temperature (50 °C)	85	27	Langmuir	Pseudo-first-order, Pseudo-second-order	22.97–32.86	Kumar et al.^54^
MNP/MWCNTs	Contact time (5–360 min), n pH (2), dosage (1.0 g/L), initial concentration (5 mg/L)	42.02		Langmuir	Pseudo-first-order, Pseudo-second-order, intra-particle diffusion	50	Lu et al.^55^
L-cysteine/Fe_3_O_4_	Contact time (5–30 min), dosage (2 g/L), initial concentration (50 mg/L), pH (3), temperature (25 °C)	34.48	58.49	Langmuir	Pseudo-first-order, Pseudo-second-order	10.4	Bagbi et al.^56^
Polyhydroxylbutyrate/CNTs	Contact time (10 min), pH (6.8), dosage (20 mg), temperature (30 °C), initial concentration (72.34 mg/L)	− 204.1	253.189	Langmuir	Pseudo-first-order, Pseudo-second-order, Elovich, fractional power	60	Bankole et al.^57^
ZnO/kaolin	Contact time (15 min), agitation speed (150 rpm), dosage 0.2 g), pH (5.84)	117.25	31.8	Jovanovic	Intra-particle diffusion, Boyd	15.02	Present study

### Reusability study

**Figure 19:**
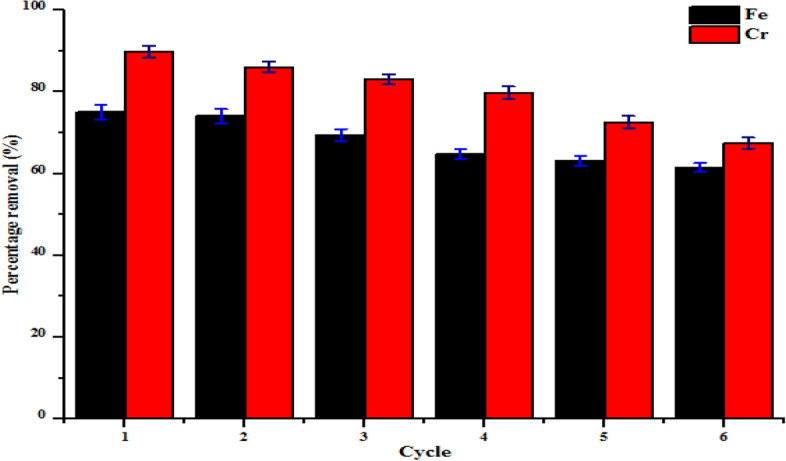
Effect of regeneration cycles on the percentage removal efficiency of Fe and Cr onto kaolin.

**Figure 20:**
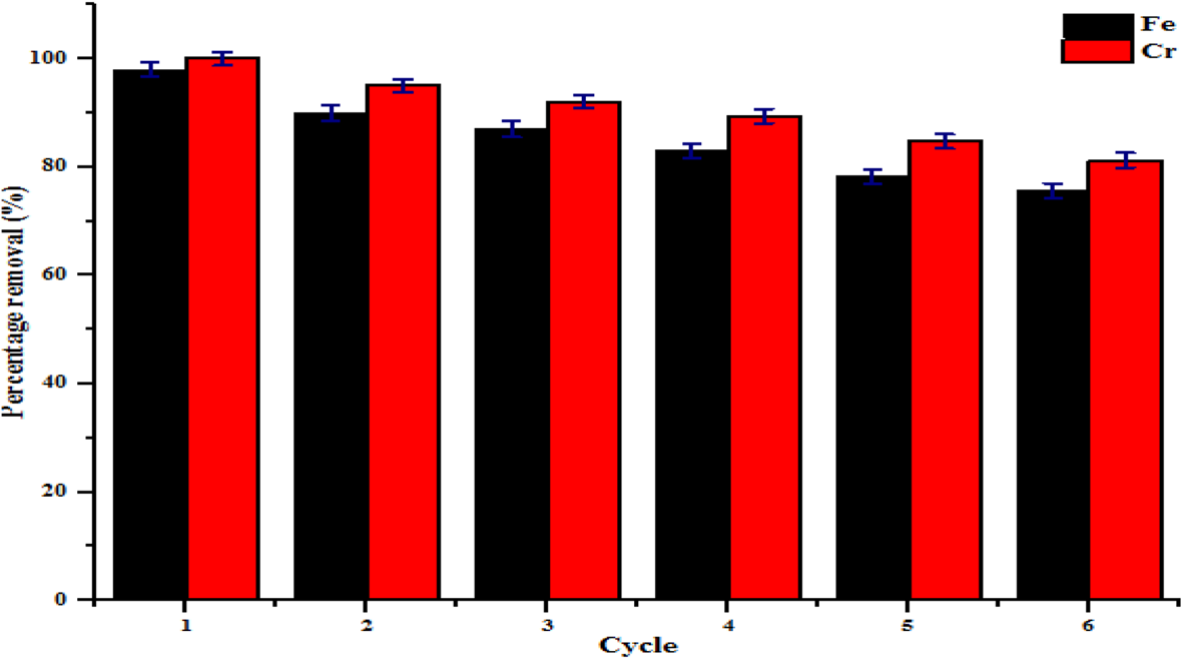
Effect of regeneration cycles on the percentage removal efficiency of Fe and Cr onto kaolin/ZnO nanoadsorbents.

The reusability and stability of the nanoadsorbents are imperative regarding the commercial application. The behaviour of kaolin and kaolin/ZnO nanocomposites in tannery wastewater in repeated adsorption–desorption cycles was carried out for six successive runs. In this research, the adsorbents were allowed to settle and collected via filtration technique and then reused by washing with 0.1 M NaOH after the adsorption of Cr(VI) and Fe(III) from tannery wastewater. The resultant sample was first oven-dried at 105 °C for 2 h and then calcined at 450 °C in a furnace for 3 h prior use in the following runs. The experiment was repeated using the optimum conditions established in batch adsorption studies. Figures 19 and 20 show the regeneration potential of kaolin and kaolin/ZnO nanocomposites on the percentage removal efficiency of Fe and Cr ions after six successive runs. It can be noticed that the amount of Fe and Cr removed decreased slightly after the second cycle (90%, 95%) compared to the first cycle (98%, 100%) and then decreased significantly after the second run for kaolin/ZnO composites. In the repeated adsorption–desorption cycles, the removal efficiencies of the heavy metals ions slightly decreased compared to the removal of metal ions in the first and second cycles. This shows that the regenerated nanocomposites showed remarkably high adsorption efficiencies for both metal ions in the first, second cycles and subsequently compared to the used of kaolin alone Generally, regeneration of nanoadsorbents is partially inhibited as a result of the loss of nanoparticles and weakness of the supporting material in the case of kaolin/ZnO mixtures. Hence, the reduction in nanoadsorbent shelf life is proportional to the adsorption removal efficiency of nanoadsorbents. The results show the good recyclability potentials of kaolin/ZnO nanocomposites for the removal of Cr and Fe ions in tannery wastewater via adsorption than kaolin alone. Yu et al.^22^ also reported excellent reusability and stability potential of 30% ZnO/biochar composite for the removal of Cr(VI) from aqueous solution after 5 cycles.

## Conclusions

The present study proves that kaolin and kaolin/ZnO nanocomposites could serve as effective adsorbents for the sequestration of Cr, Fe, COD, BOD and chloride from tannery wastewater. The adsorbents were analysed by XRD, FTIR, BET, HRTEM, SAED, EDX and XPS. The adsorption process was found to be dependent on the contact time, adsorbent dosage and temperature. Kaolin/ZnO composites exhibited excellent adsorptive behaviour under the applied conditions than kaolin alone based on the differences in surface area. The optimum conditions for the removal of Cr, Fe, COD, BOD and chloride varies and the differences were linked to the nature of the pollutants in tannery wastewater. The equilibrium adsorption data could be best described by Jovanovic adsorption isotherm model. Adsorption kinetic results indicated that both intra-particle and bulk diffusion were the rate-determining steps. Thermodynamic parameters revealed that the adsorption processes were endothermic in nature and non-spontaneous. The results show the practical applicability of kaolin/ZnO nanocomposites for the adsorption of pollutants from tannery wastewater thus making the adsorbent a promising candidate in the field of water and wastewater management.
